# Mfsd2a^+^ hepatocytes repopulate the liver during injury and regeneration

**DOI:** 10.1038/ncomms13369

**Published:** 2016-11-18

**Authors:** Wenjuan Pu, Hui Zhang, Xiuzhen Huang, Xueying Tian, Lingjuan He, Yue Wang, Libo Zhang, Qiaozhen Liu, Yan Li, Yi Li, Huan Zhao, Kuo Liu, Jie Lu, Yingqun Zhou, Pengyu Huang, Yu Nie, Yan Yan, Lijian Hui, Kathy O. Lui, Bin Zhou

**Affiliations:** 1 Key Laboratory of Nutrition and Metabolism, Institute for Nutritional Sciences, Shanghai Institutes for Biological Sciences, Graduate School of the Chinese Academy of Sciences, Chinese Academy of Sciences, Shanghai, 200031, China; 2 State Key Laboratory of Cell Biology, CAS Center for Excellence in Molecular Cell Science, Institute of Biochemistry and Cell Biology, Shanghai Institutes for Biological Sciences, Chinese Academic of Sciences, Yueyang Road 320, A2112, Shanghai 200031, China; 3 Department of Gastroenterology, Shanghai 10th People's Hospital, Tongji University School of Medicine, Shanghai 200072, China; 4 School of Life Science and Technology, Shanghai Tech University, Shanghai 201210, China; 5 State Key Laboratory of Cardiovascular Disease, Fuwai Hospital, National Center for Cardiovascular Disease, Chinese Academy of Medical Sciences and Peking Union Medical College, Beijing 100037, China; 6 Department of Cardiology, Zhongshan Hospital, Fudan University, Shanghai 200032, China; 7 Department of Chemical Pathology, Li Ka Shing Institute of Health Sciences, The Chinese University of Hong Kong, Prince of Wales Hospital, Shatin, Hong Kong SAR 999077, China; 8 Institute of Neuroscience, State Key Laboratory of Neuroscience, CAS Center for Excellence in Brain Science and Intelligence Technology, Shanghai Institutes for Biological Sciences, Chinese Academy of Sciences, Shanghai 200031, China; 9 Key Laboratory of Regenerative Medicine of Ministry of Education, Institute of Aging and Regenerative Medicine, Jinan University, Guangzhou 510632, China

## Abstract

Hepatocytes are functionally heterogeneous and are divided into two distinct populations based on their metabolic zonation: the periportal and pericentral hepatocytes. During liver injury and regeneration, the cellular dynamics of these two distinct populations remain largely elusive. Here we show that major facilitator super family domain containing 2a (Mfsd2a), previously known to maintain blood–brain barrier function, is a periportal zonation marker. By genetic lineage tracing of Mfsd2a^+^ periportal hepatocytes, we show that Mfsd2a^+^ population decreases during liver homeostasis. Nevertheless, liver regeneration induced by partial hepatectomy significantly stimulates expansion of the Mfsd2a^+^ periportal hepatocytes. Similarly, during chronic liver injury, the Mfsd2a^+^ hepatocyte population expands and completely replaces the pericentral hepatocyte population throughout the whole liver. After injury recovery, the adult liver re-establishes the metabolic zonation by reprogramming the Mfsd2a^+^-derived hepatocytes into pericentral hepatocytes. The evidence of entire zonation replacement during injury increases our understanding of liver biology and disease.

## Abstract (web summary)

Hepatocytes are highly specialized cells and their fate is determined by their position in the liver as either periportal or perivenous hepatocytes. Here, Pu *et al*. show through genetic lineage tracing for Mfsd2 that periportal hepatocytes proliferate and reprogram into pericentral hepatocytes during liver regeneration and injury.

## Graphical abstract

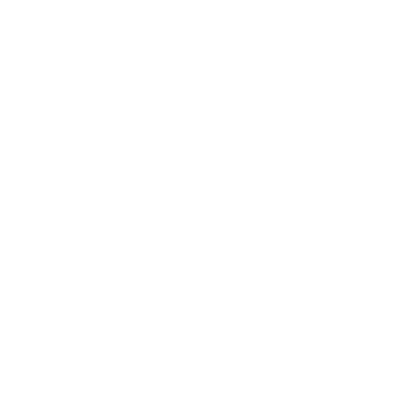

The adult liver possesses a unique regenerative capacity to maintain homeostasis. Unraveling the source of newly formed hepatocytes during liver homeostasis and regeneration remains an important task in liver biology^1^,^2^,^3^. It has been reported that hepatocyte stem or precursor cells can give rise to new hepatocytes after injury^4^. More recently, by virtue of lineage tracing studies, most, if not all, new hepatocytes are found to be derived from pre-existing hepatocytes^2^,^5^,^6^. It is generally accepted that the main cellular mechanism for hepatocyte replenishment is mediated through self-expansion. However, hepatocytes in the liver are not uniform, as they exhibit molecular and metabolic heterogeneity based on their location within the hepatic lobule, which is known as hepatic zonation^7^,^8^. At least two distinct metabolic zonations have been reported: the periportal (PP) and pericentral (PC) hepatocytes^9^,^10^. The liver cell plate consists of 15–25 hepatocytes that extend from the portal triad to the hepatic venule^11^. Of which, about six PP hepatocytes surround the portal triad (zone 1), about three PC hepatocytes are found proximal to the central triad (zone 3) and about six to seven hepatocytes located in between (zone 2)^11^. In fact, the PP region was located in zone 1 and the portal vein side of zone 2, whereas the PC region was located in zone 3 and the central vein side of zone 2 (Supplementary Fig. 1)^11^. Hepatocytes along the portal–central axis of the liver lobule have distinct yet complementary metabolic functions and many critical enzymes or proteins are preferentially expressed in either the PP or PC hepatocyte subpopulation^12^. However, how the hepatocyte heterogeneity is maintained in the liver lobule during homeostasis or regeneration after injury remains elusive. A recent study revealed that a subset of PP hybrid cells containing both ductal cells and hepatocytes replenish the liver after chronic injury without giving rise to tumour^13^. Using double recombination technology, Karin and colleagues^13^ showed that Sox9^+^ hybrid hepatocytes undergo extensive proliferation during chronic injuries without giving rise to hepatocellular carcinoma. In addition, another report showed that Axin2^+^ hepatocytes adjacent to the central vein proliferate and expand up to 30% of total area of the entire liver during homeostasis^14^. Despite the fact distinct cell populations involved in liver regeneration are identified, how the PP and PC hepatocyte populations respond to injury and, if there is a repopulation, priority of a particular hepatocyte population during liver regeneration remain enigmatic. Major facilitator super family domain containing 2a (Mfsd2a) is expressed by the brain blood vessels and is critical for the formation and function of the blood–brain barrier (BBB)^15^,^16^. We found that Mfsd2a is also expressed in PP hepatocyte and we used genetic lineage tracing to label these Mfsd2a^+^ hepatocytes. During liver homeostasis, the PP hepatocyte population decreases. Nevertheless, liver regeneration induced by partial hepatectomy (PH) and chronic liver injury significantly stimulates expansion of the Mfsd2a^+^ PP hepatocytes, replacing the PC hepatocyte population. The evidence of organ-wide cellular compensation by PP hepatocytes post liver injury could be of relevance to liver biology and diseases.

## Results

### Identification of Mfsd2a as a marker of PP hepatocytes

**Figure 1:**
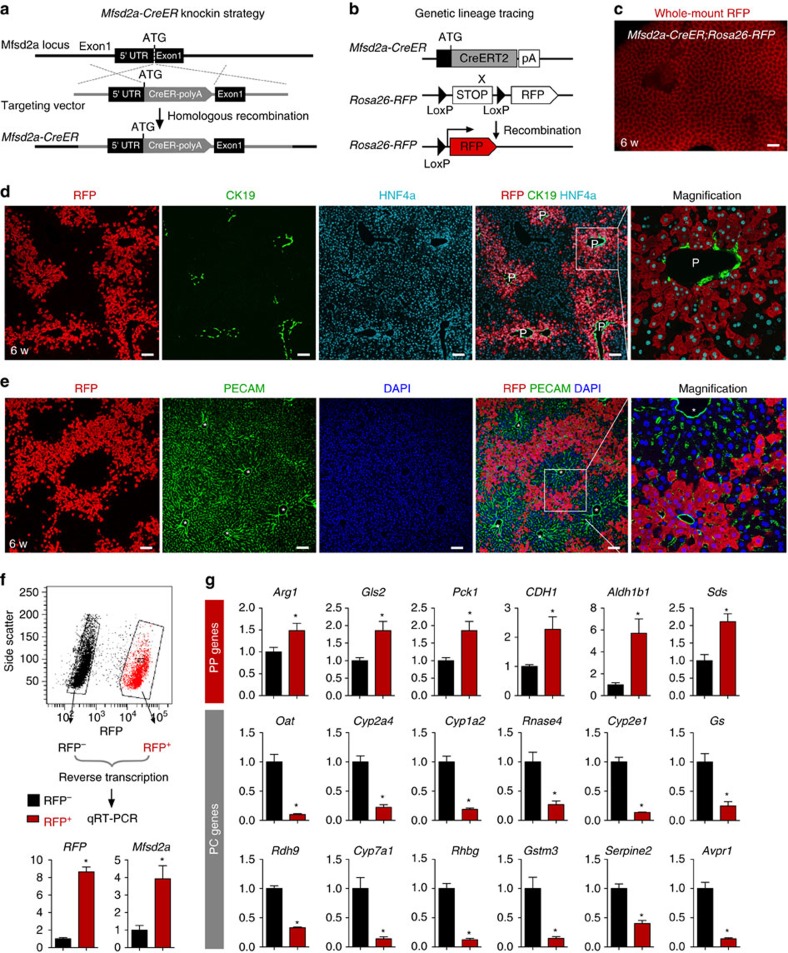
PP hepatocytes labelled by Mfsd2a-CreER. Schematic figures showing (**a**) our knock-in strategy for *Mfsd2a-CreER* allele using CRISPR/Cas9 by homologous recombination and (**b**) genetic lineage tracing strategy for Mfsd2a^+^ hepatocytes by Cre-LoxP recombination in Mfsd2a^+^ hepatocytes. (**c**) Whole-mount fluorescence view of the adult liver from 6-week-old *Mfsd2a-CreER;Rosa26-RFP* mice. Tamoxifen was induced at 2 days before analysis. Scale bar, 1 mm. (**d**) Immunostaining for RFP, CK19 and HNF4a on liver sections shows Mfsd2a-expressing hepatocytes in PP zone (P). Scale bars, 100 μm. (**e**) Immunostaining for RFP, PECAM and 4,6-diamidino-2-phenylindole (DAPI) on liver sections shows Mfsd2a-expressing hepatocytes in the PP zone but not PC zone (*). Scale bars, 100 μm. (**f**) Isolation of RFP^−^ and RFP^+^ cells by flow cytometry followed by quantitative RT–PCR (qRT–PCR) analysis for expression of *RFP* and *Mfsd2a*. Expression level of genes in the RFP^−^ cells was set as 1. Error bars are s.e.m. of the mean for all the quantification in this study. (**g**) Expression of PP and PC genes detected by qRT–PCR. The *x* axis denotes RFP^−^ (black) and RFP^+^ (red) groups, and the *y* axis denotes fold induction.. *P<0.05; *n*=3, two-tailed unpaired *t*-test. Each immunostaining image is a representative of four individual samples.

We first generated *Mfsd2a-CreER* for studying BBB (Fig. 1a) and confirmed that *Mfsd2a-CreER* was specifically expressed by the brain vascular endothelial cells (Supplementary Fig. 2a). Our results are in line with previous reports^15^,^16^. We then crossed *Mfsd2a-CreER* with the *Rosa26-RFP* reporter line, to irreversibly label the Mfsd2a-expressing cells and their descendants with the genetic marker red fluorescent protein (RFP; Fig. 1b). When tamoxifen was administered to induce recombination at the embryonic stage, *Mfsd2a-CreER* specifically labelled blood vessels in the brain but not in other organs including the heart, lung, kidney and liver (Supplementary Fig. 2b–e), indicating its BBB specificity^15^,^16^. When tamoxifen was administered in the *Mfsd2a-CreER;Rosa26-RFP* mice at the adult stage, we unexpectedly observed a unique ‘lattice' pattern in the liver (Fig. 1c). We then performed immunostaining for the genetic marker RFP with the biliary epithelial cell marker CK19 and hepatocyte marker HNF4a on liver sections of the *Mfsd2a-CreER;Rosa26-RFP* mice. Our results showed that *Mfsd2a-CreER* specifically labelled PP hepatocytes and very sparsely labelled PC hepatocytes (<1% PC hepatocytes; Fig. 1d). By reducing to one-tenth of tamoxifen dosage, we found that *Mfsd2a-CreER* specifically labelled PP hepatocytes only but not the PC hepatocytes (Supplementary Fig. 3). Although induction of tamoxifen in *Mfsd2a-CreER;Rosa26-RFP* mice labelled most PP hepatocytes, few RFP^+^ cells were detected without tamoxifen treatment (Supplementary Fig. 4). Similarly, the results from *in situ* hybridization of *Mfsd2a* confirmed PP expression pattern (Supplementary Fig. 5a). Furthermore, we performed immunostaining of oestrogen receptor (ESR, as a surrogate for Mfsd2a expression) and RFP on liver sections collected from *Mfsd2a-CreER;Rosa26-RFP* mice within 48 h after tamoxifen induction. We found that ESR and RFP were co-localized in most PP hepatocytes (Supplementary Fig. 5b). Immunostaining for RFP and other non-hepatocyte lineage-specific markers showed that *Mfsd2a-CreER* did not label biliary epithelial cells, hepatic stellate cells, vascular endothelial cells, smooth muscle cells or fibroblasts in the liver (Fig. 1e and Supplementary Fig. 6). We next used the *Mfsd2a-CreER;Rosa26-RFP* mice to isolate PP and PC hepatocytes for gene expression analysis. We first confirmed that we were able to purify Mfsd2a^+^ hepatocytes via flow cytometry by virtue of the RFP reporter expression (Fig. 1f). We then measured previously reported PP and PC zonation genes in the purified RFP^+^ and RFP^−^ populations^12^. Expression levels of the PP zonation genes were significantly higher in the RFP^+^ than the RFP^−^ population, whereas expression levels of the PC zonation genes were significantly lower in the RFP^+^ than the RFP^−^ population (Fig. 1g). Taken together, our results showed that Mfsd2a was specifically expressed by the PP hepatocytes of adult liver and the *Mfsd2a-CreER* mouse line could be used to study the cell fate of PP hepatocyte population during liver regeneration *en masse*.

### Reduced Mfsd2a^+^ PP hepatocytes are found during liver homeostasis

**Figure 2:**
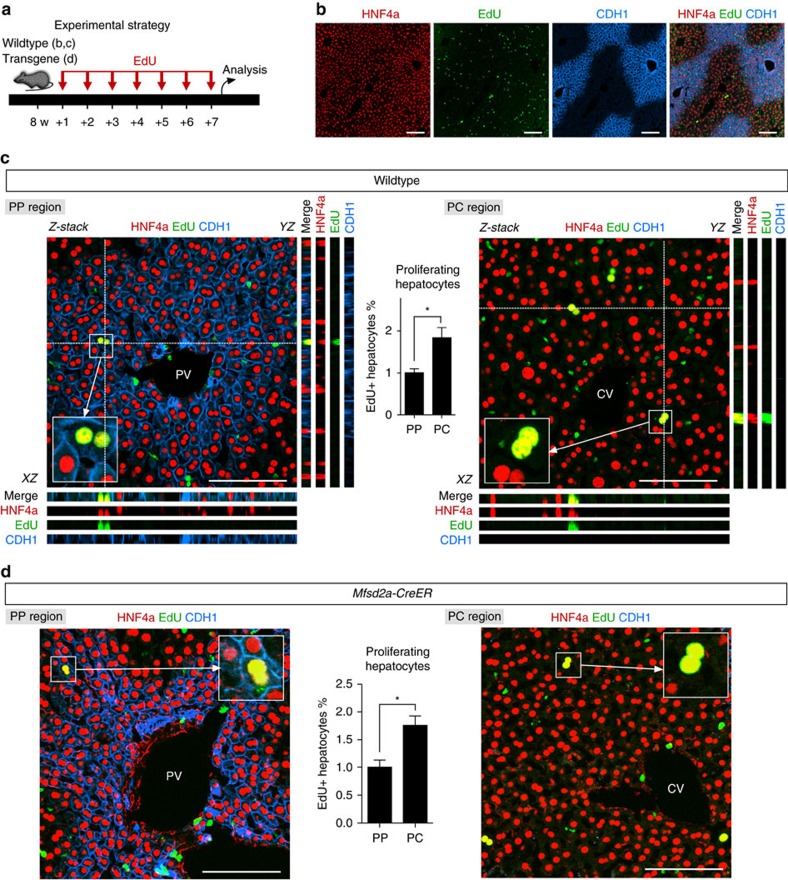
PC hepatocytes proliferate faster than PP hepatocytes. (**a**) Schematic figure showing time points for EdU injection. (**b**) Immunostaining for EdU, hepatocyte marker HNF4a and PP zonation marker CDH1 on liver sections of wild-type mice. Scale bars, 100 μm. (**c**) *Z*-stack images of PP and PC regions of liver sections. *XZ* and *YZ* indicate signals from dotted lines on *Z*-stack images. Inserts are magnified images. Quantification of percentage of EdU^+^ hepatocytes on PP and PC regions is shown in the middle panel. Scale bars, 100 μm. (**d**) Immunostaining for HNF4a, EdU and CDH1 on liver sections of Mfsd2a-CreER line. Quantification of percentage of EdU^+^ hepatocytes on PP and PC regions is shown in the middle panel. *P<0.05; *n*=6; two-tailed unpaired *t*-test; Scale bars, 100 μm. Error bars are s.e.m. of the mean. Each image is a representative of six individual samples.

**Figure 3:**
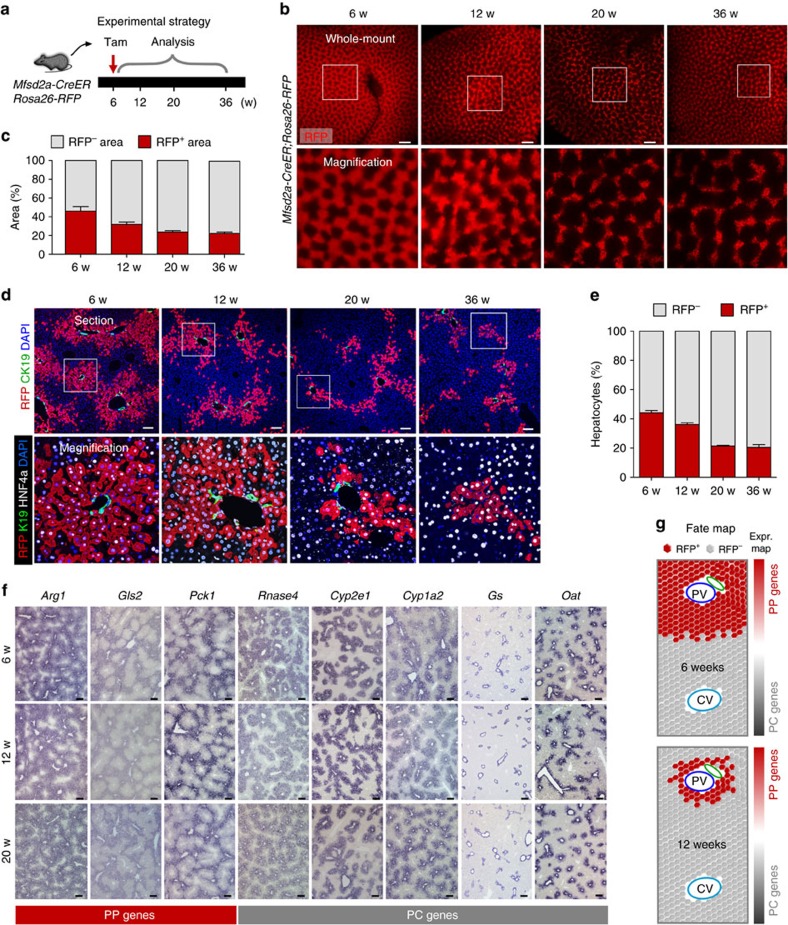
PP hepatocytes reduced during liver homeostasis. (**a**) Schematic figure showing experimental strategy for tamoxifen treatment (Tam) and tissue analysis at the indicated time points after Tam. (**b**) Whole-mount fluorescence view of *Mfsd2a-CreER;Rosa26-RFP* livers collected at the indicated time points. Scale bars, 1 mm. (**c**) Quantification of the percentage of RFP^+^ and RFP^−^ area. *n*=4. Error bars are s.e.m. of the mean. (**d**) Immunostaining for RFP, CK19 and HNF4a on liver sections shows that RFP^+^ hepatocytes remains in the PP regions during homeostasis. Scale bars, 100 μm. (**e**) Quantification of the percentage of RFP^+^ hepatocytes in the liver. (**f**) *In situ* hybridization of PP or PC genes on liver sections from mice at 6–20 weeks old. Scale bars, 500 μm. (**g**) Schematic figure showing PP hepatocytes reduced during liver homeostasis, whereas expression (Expr.) of zonation genes is maintained. Each image is a representative of four individual samples.

Although the PP and PC hepatocyte populations are distinct in terms of their molecular profiles and functions^7^,^8^,^12^, we sought to address their repopulation capacity during homeostasis. We first analysed the proliferation of PP and PC hepatocytes by 5-ethynyl-2′-deoxyuridine (EdU) incorporation assay in the wild-type mice (Fig. 2a) by adopting similar experimental strategies as previously described^14^,^17^. Immunostaining for EdU, HNF4a (hepatocyte marker) and CDH1 (PP zonation marker) showed that a subset of hepatocytes (EdU^+^HNF4a^+^) were proliferating in both CDH1^+^ and CDH1^−^ regions (Fig. 2b). Quantification of proliferating hepatocytes in both PP and PC regions showed a significantly higher number of proliferating hepatocytes in PC region than that in PP regions (percentage of EdU^+^ hepatocytes: 1.76±0.17% versus 1.01±0.092%, *n*=6; Fig. 2c). Similarly, we found more proliferating hepatocytes in PC region than in PP region of *Mfsd2a-CreER* transgenic mice (percentage of EdU^+^ hepatocytes: 1.84±0.24% versus 0.99±0.12%, *n*=6; Fig. 2d). We next performed genetic lineage-tracing experiments using *Mfsd2a-CreER;Rosa26-RFP* by administering tamoxifen at 6 weeks and performing analysis at later stages (Fig. 3a). Whole-mount fluorescence views of livers collected at 6-, 12- and 20-week-old mice showed a significant regression of RFP^+^ area with time, indicating that those unlabelled hepatocytes expanded and replaced some of the Mfsd2a-derived PP hepatocytes (*n*=4; Fig. 3b,c). However, there was no significant regression of the RFP^+^ area from 20 to 36 weeks (Fig. 3b,c). Immunostaining for RFP and CK19 on liver sections demonstrated that prelabelled PP hepatocytes reduced from 8–9 to 3–4 cell layers from week 6 to 20, which maintained thereafter till week 36 (Fig. 3d). Quantification for the percentage of RFP^+^ hepatocytes among all hepatocytes showed a significant reduction in RFP^+^ hepatocytes at 6- to 20-week-old mice (Fig. 3e), indicating that unlabelled hepatocytes competed over the labelled PP population during homeostasis. There is no significant reduction in RFP^+^ hepatocytes at 20- to 36-week-old mice (Fig. 3e).

Although the fate-mapping data suggested that there was an expansion priority of different zonation in normal liver, we asked whether the zonation gene expression profile remained unchanged in 6- to 20–week-old mice. Our *in situ* hybridization experiments showed that the expression patterns of PP genes (*Arg1*, *Gls2* and *Pck1*) and PC genes (*Rnase4*, *Cyp2e1*, *Cyp1a2*, *GS* and *Oat*) were similar among different ages (Fig. 3f). Taken together, our fate-mapping data together with metabolic gene expression profiles demonstrated that a significant portion of the Mfsd2a^+^ PP hepatocytes were replaced by unlabelled hepatocytes (probably PC hepatocytes) in the liver during homeostasis and these newly positioned hepatocytes were reprogrammed into the PP hepatocyte fate with expression of PP genes (Fig. 3g).

### Mfsd2a^+^ PP hepatocytes expand during liver regeneration

**Figure 4:**
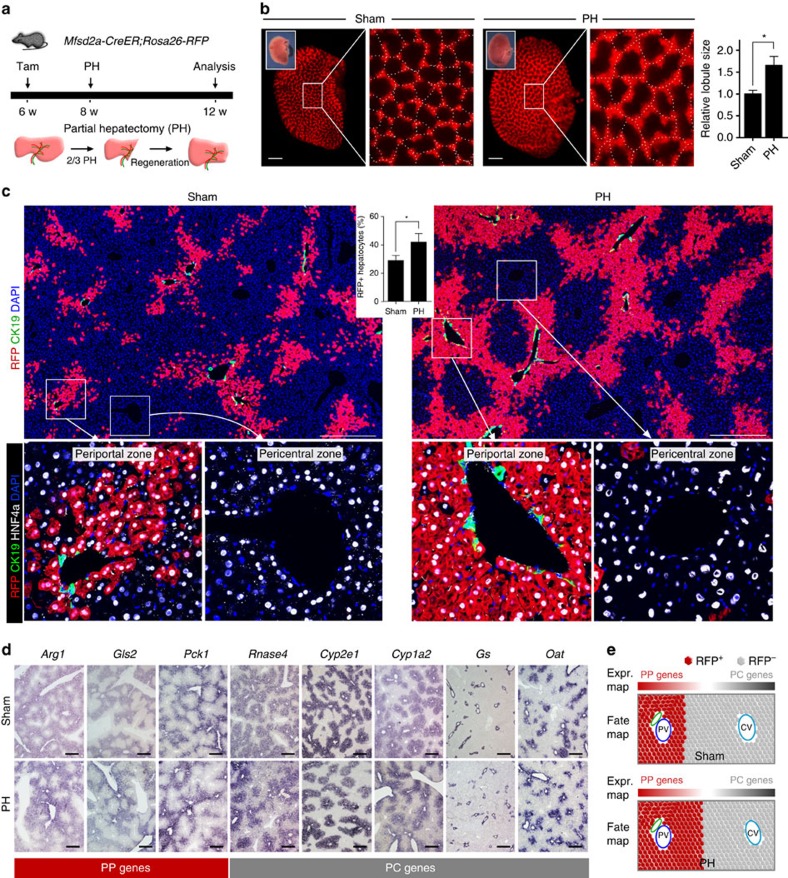
PP hepatocytes expand during liver regeneration. (**a**) Schematic figure showing experimental strategy for tamoxifen induction and PH of *Mfsd2a-CreER;Rosa26-RFP* mice. (**b**) Whole-mount fluorescence view of sham-operated or PH liver. Inserts indicate the bright-field view of the same liver. Dotted lines demarcate liver lobule. Quantification of the liver lobule size based on sham-operated control (set as 1). *P<0.05; *n*=4; two-tailed unpaired *t*-test. Error bars are s.e.m. of the mean. Scale bars, 1 mm. (**c**) Immunostaining for RFP, CK19 and HNF4a on liver sections shows expansion of RFP+ hepatocytes during liver regeneration compared with the sham control. Inserts indicate quantification of the percentage of RFP^+^ hepatocytes in sham or PH livers. *P<0.05; *n*=4; two-tailed unpaired *t*-test. Scale bars, 500 μm. (**d**) *In situ* hybridization of PP and PC genes in sham or PH livers. Scale bars, 500 μm. (**e**) Schematic figure showing PP hepatocytes expand during liver regeneration, whereas expression (Expr.) of zonation genes is maintained. Each image is a representative of four individual samples.

We next asked whether liver regeneration alters the repopulation priority of the PP and PC hepatocytes. The adult liver regenerated completely within 2 weeks after two-third PH (Fig. 4a). Intriguingly, whole-mount fluorescence view of the *Mfsd2a-CreER;Rosa26-RFP* liver showed that RFP^+^ lattice pattern remained and the size of lattice increased after injury (Fig. 4b, 1.66 times of size compared with the sham-operated group, *n*=4), indicating that each lobule served as a basic unit for self-expansion during liver regeneration. Immunostaining for RFP and CK19 on liver sections confirmed that liver lobule expanded in size with maintenance of PP and PC hepatocytes in this basic unit without a significant intermixing of two hepatocyte populations (Fig. 4c). Moreover, RFP^+^ hepatocytes in each lobule expanded in size after PH compared with that of the sham-operated liver (Fig. 4c,d), demonstrating an efficient expansion of the PP population during liver regeneration compared with the same population in sham-operated group (percentage of RFP^+^ hepatocytes: 42.62±6.05% versus 29.34±3.65%, respectively, *n*=4). The differential compartmentalization of two hepatocyte populations suggested that the hepatocytes are heterogeneous in compensatory growth during homeostasis and regeneration. In addition, we also observed that the metabolic zonation remained roughly the same in the liver lobule during regeneration (Fig. 4e).

During liver regeneration, we examined the cell proliferation rate at early and late stages after PH. We induced PH 2 weeks following treatment of tamoxifen in *Mfsd2a-CreER;Rosa26-RFP* mice at 6 weeks old. We then collected liver samples at day 2 (early stage) and week 4 (late stage) post PH, and examined hepatocyte proliferation by immunostaining for RFP and Ki67 on liver sections (Supplementary Fig. 7). Our results showed that there were significantly more RFP^+^Ki67^+^ hepatocytes in the PP than PC region at day 2 post PH. Moreover, there were significantly less RFP^+^Ki67^+^ hepatocytes at week 4 than day 2 post PH, suggesting that proliferation was reduced to baseline by 4 weeks after PH (Supplementary Fig. 7).

**Figure 5:**
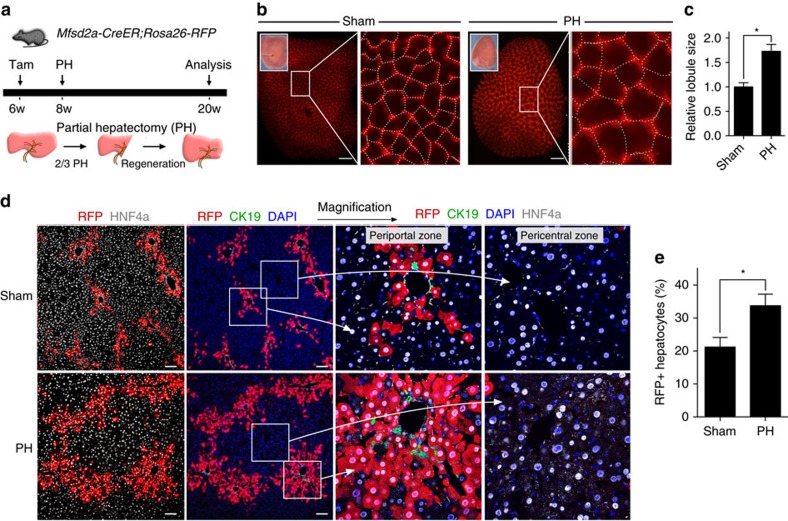
Lineage tracing of Mfsd2a-derived hepatocytes during liver regeneration. (**a**) Schematic figure showing experimental strategy for tamoxifen induction and PH of *Mfsd2a-CreER;Rosa26-RFP* mice. (**b**) Whole-mount fluorescence view of sham-operated or PH liver. Inserts indicate the bright-field view of the same liver. Dotted lines define liver lobule. Scale bars, 1 mm. (**c**) Quantification of the liver lobule size based on sham-operated control (set as 1). *P<0.05; *n*=4; two-tailed unpaired *t*-test. (**d**) Immunostaining for RFP, CK19 and HNF4a on liver sections showing expansion of RFP^+^ hepatocytes during liver regeneration compared with the sham control. Scale bars, 100 μm. (**e**) Quantification of the percentage of RFP^+^ hepatocytes in sham or PH livers. *P<0.05; *n*=4; two-tailed unpaired *t*-test. Error bars are s.e.m. of the mean. Each image is a representative of four individual samples.

We also performed long-term lineage tracing on RFP^+^ cells after PH (Fig. 5a). By collecting liver samples at week 12 after PH, we found that the size of liver lobule was still larger in the PH than sham group (1.73 times, *n*=4; Fig. 5b,c). Immunostaining for RFP, HNF4a and CK19 showed that there were more RFP^+^HNF4a^+^ hepatocytes in the PP than PC region (Fig. 5d). Quantification of labelled cells showed that there was a significant increase in the percentage of RFP^+^ hepatocytes after PH compared with the sham-operated group (percentage of RFP^+^ hepatocytes: 33.74±3.52% versus 21.21±2.91%, respectively, *n*=4; Fig. 5e). Together, our data suggested that PP hepatocytes might undergo more robust proliferation shortly after PH and the expanded Mfsd2a^+^ hepatocytes remained for a long time in the liver after PH.

### Complete replacement of PC hepatocytes by PP hepatocytes during liver injury

**Figure 6:**
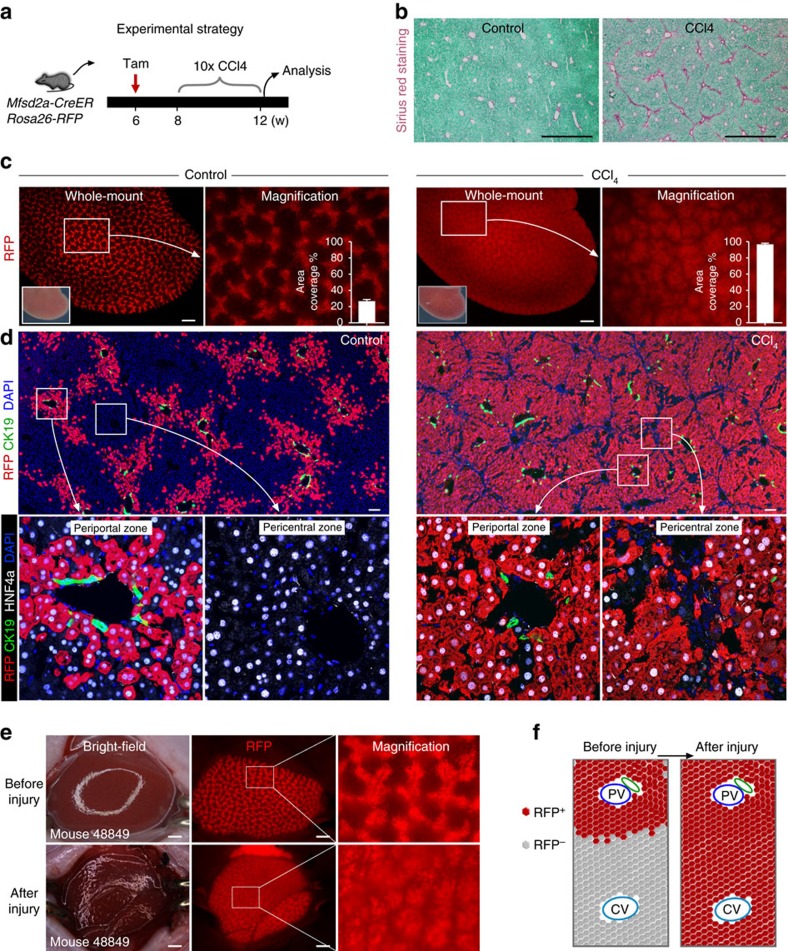
PP hepatocytes expand and replace almost all hepatocytes in the liver lobule after injury. (**a**) Schematic figure showing experimental strategy for tamoxifen induction and CCl_4_ treatment. (**b**) Sirius red staining images showing robust fibrotic responses in CCl_4_-treated group, compared with oil-treated group (control). Scale bars, 1 mm. (**c**) Whole-mount fluorescence view of *Mfsd2a-CreER;Rosa26-RFP* livers from control (left) and CCl_4_ (right)-treated groups. *n*=4. Error bars are s.e.m. of the mean. Scale bars, 1 mm. (**d**) Immunostaining for RFP, CK19 and HNF4a on *Mfsd2a-CreER;Rosa26-RFP* liver sections of control and CCl_4_-treated mice. Scale bars, 100 μm. (**e**) Sequential whole-mount fluorescence view of the same liver from individual mouse before injury and at week 4 after chronic injury. Scale bars, 1 mm. (**f**) Schematic figure showing expansion of PP hepatocytes after injury. CV, central vein; PV, portal vein. Each image is a representative of four individual samples.

We next asked whether chronic injury would alter the compartmentalization of PP and PC hepatocytes. The *Mfsd2a-CreER;Rosa26-RFP* mice were treated with CCl_4_ to induce chronic injury (Fig. 6a), which was confirmed by notable fibrosis (Fig. 6b). By collecting livers from oil-treated control mice or CCl_4_-treated mice, we repeatedly observed the ‘lattice' pattern of RFP^+^ hepatocytes in the control liver and expansion of RFP^+^ hepatocytes in the entire liver of CCl_4_-treated mice (Fig. 6c). Immunostaining for RFP and CK19 showed that pre-labelled PP hepatocytes expanded in every hepatic lobule throughout the entire liver (Fig. 6d). Magnification of the PC regions also showed that hepatocytes in the PC regions were RFP^+^, suggesting that the PC hepatocytes were replaced by Mfsd2a-derived PP hepatocytes during chronic injury (Fig. 6d). Notably, we observed very few RFP^−^ hepatocytes in both PP and PC zones, and our quantification results confirmed that there was no difference in percentage of RFP^−^ hepatocytes in both regions, indicating that the unlabelled cells were likely to be attributed to the labelling efficiency of CreER. We next examined proliferation of hepatocytes after CCl_4_ treatment. Immunostaining for Ki67 showed that a significantly more Ki67^+^ hepatocytes in the PP than PC region at day 2 after CCl_4_ injection and the number of proliferating RFP^+^ hepatocytes reduced substantially after 4 weeks of CCl_4_ treatment (chronic injury; Supplementary Fig. 8).

In the fate-mapping study, few hepatocytes were labelled in the absence of tamoxifen treatment (Supplementary Fig. 9). We also compared the proliferation rate of few ‘leaky' RFP^+^ hepatocytes at day 2 after single CCl_4_ injection and at week 4 after chronic CCl_4_ treatment. Immunostaining for Ki67 showed that there was no difference in proliferation between these ‘leaky' RFP^+^ hepatocytes and unlabelled PP hepatocytes at day 2 after CCl_4_ injection and at week 4 after chronic CCl_4_ treatment (Supplementary Fig. 10). At week 4 after chronic injury, these ‘leaky' RFP^+^ hepatocytes contributed minimally to the whole population of hepatocytes (Supplementary Figs 9e and 10c), compared with the tamoxifen-induced RFP^+^ hepatocytes that repopulated the entire liver after CCl_4_ treatment (Fig. 6d). These data suggested that PP hepatocytes repopulated the liver after CCl_4_-induced chronic injury.

To visualize this expansion from the beginning to the end of injury in the same mouse liver, we collected whole-mount fluorescence images before and after injury from the same liver of *Mfsd2a-CreER;Rosa26-RFP* mouse. We found that the ‘lattice' pattern turned into massive RFP^+^ signals throughout the entire liver after injury (Fig. 6e). To further understand the cellular dynamics at the intermediate stage, we collected livers at week 2 after chronic injury when fibrotic tissues started to form (Supplementary Fig. 11a). Whole-mount view showed RFP^+^ PP hepatocytes expanded to most regions (Supplementary Fig. 11b). Immunostaining for RFP, CK19 and HNF4a showed that most PC hepatocytes were ablated at week 2 after chronic injury and RFP^+^ hepatocytes reached the periphery of PC region but not the central vein (Supplementary Fig. 11c), suggesting that PP hepatocytes underwent compensatory expansion to replace the PC hepatocyte population.

We next asked whether the injury induced by CCl_4_ injection extended into the zone containing Mfsd2a^+^ hepatocytes. By immunostaining for the surrogate marker of Mfsd2a, ESR, and HNF4a on liver sections collected at day 2 after CCl_4_ injection, we found that the extent of injury (defined by HNF4a^+^ hepatocyte cell death) did not reach the zone containing Mfsd2a-expressing hepatocytes (ESR^+^; Supplementary Fig. 12a). By immunostaining for COL3A1 and ESR on liver sections collected after 4 weeks of CCl_4_ treatment, we found that hepatocytes reappeared in the PC region and COL3A1^+^ fibrotic tissues (as injury region) did not extend into the zone containing Mfsd2a-expressing hepatocytes (ESR^+^; Supplementary Fig. 12b). These data suggested that CCl_4_-induced injury mainly targeted those Mfsd2a^−^ hepatocytes but rarely, if any, the Mfsd2a^+^ PP hepatocytes.

**Figure 7:**
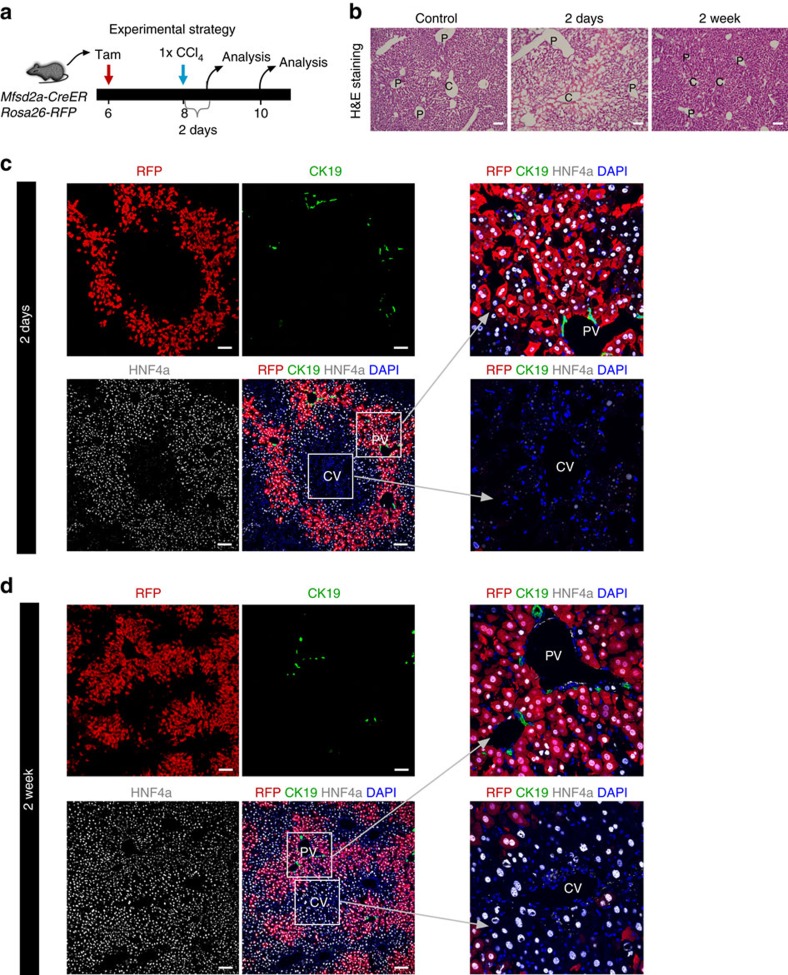
Lineage tracing of Mfsd2a^+^ hepatocytes after single injection of CCl_4_. (**a**) Schematic figure showing strategy for tamoxifen induction (Tam), CCl_4_ treatment and time points for tissue analysis. (**b**) Haematoxylin and eosin staining of liver sections collected at day 2 or week 2 after after single CCl_4_ injection. C, central vein; P, portal vein. Scale bars, 100 μm. (**c**,**d**) Immunostaining for RFP, CK19 and HNF4a on liver sections collected at day 2 (**c**) and week 2 (**d**) after single CCl_4_ injection. Scale bars, 100 μm. Each image is a representative of four individual samples.

We next asked whether a single dose of CCl_4_ injection led to expansion of Mfsd2a^+^ PP hepatocytes after 2 weeks' recovery. *Mfsd2a-CreER;Rosa26-RFP* mice were treated with tamoxifen at 6 weeks old and injected with CCl_4_ at 8 weeks old (Fig. 7a). By examining liver samples using haematoxylin and eosin staining and immunostaining, we found severe liver injury at day 2 when almost no hepatocyte could be detected at the PC region (Fig. 7b,c). Haematoxylin and eosin staining and immunostaining for HNF4a showed that the liver had recovered 2 weeks after CCl_4_ injection. The percentage of RFP^+^ hepatocytes increased at week 2 compared with day 2 after injury (Fig. 7d). Unlike chronic injury with multiple CCl_4_ injections (Fig. 6d), Mfsd2a^+^ hepatocytes did not expand to replace the entire liver lobule (Fig. 7d). Therefore, our data suggested that complete hepatic zonation replacement happened only after chronic but not acute injury following CCl_4_ treatment. Altogether, our results demonstrated that the Mfsd2a-derived PP hepatocyte population completely replaced the PC hepatocyte population in the entire liver during CCl_4_-induced chronic injury (Fig. 6f).

### Reprogramming of metabolic zonation during liver regeneration

**Figure 8:**
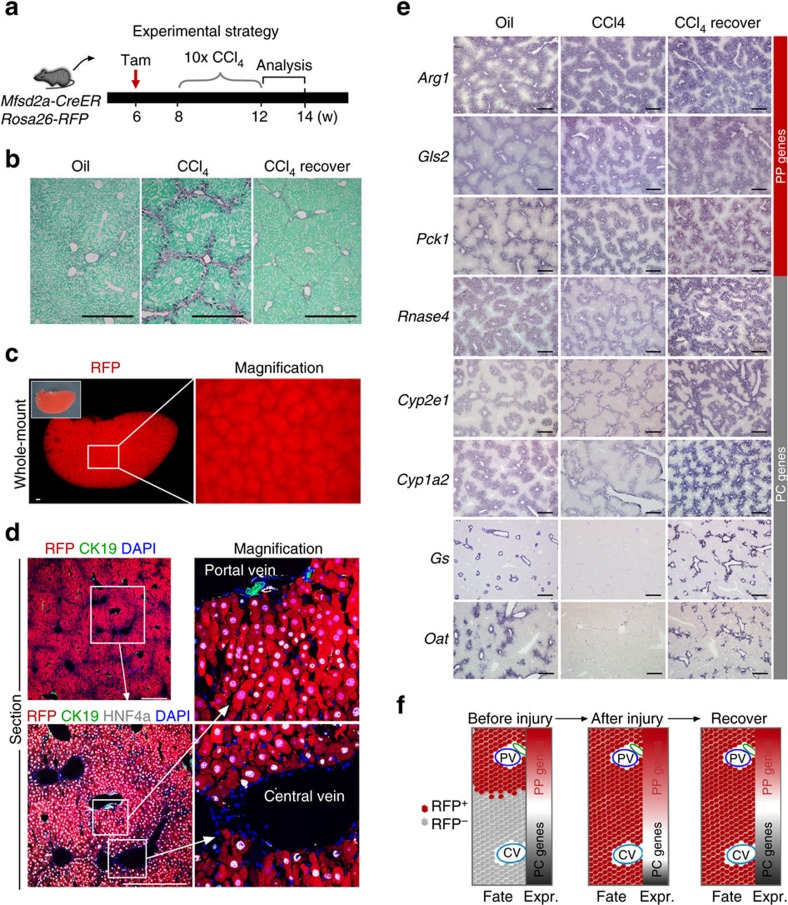
Restoration of PC gene expression during recovery. (**a**) Schematic figure showing experimental strategy at the indicated time points. (**b**) Images of liver sections stained with Sirius red. Scale bars, 500 μm. (**c**) Whole-mount fluorescence view of liver of CCl_4_-treated mice during recovery. Scale bars, 500 μm. (**d**) Immunostaining for RFP, CK19 and HNF4a showing RFP^+^ PC and PP hepatocytes in the recovered liver. Scale bars, 500 μm. (**e**) Images of *in situ* hybridization showing recovered PC gene expression during recovery. Scale bars, 500 μm. (**f**) A cartoon figure showing expansion of PP hepatocytes throughout the entire liver after injury and recovery, whereas some PC genes (grey) were reduced after injury and restored during recovery. Each image is a representative of four individual samples.

We examined liver metabolic zonations during liver regeneration by examining the gene expression profile in injured and recovered liver (Fig. 8a). At week 2 after removal of CCl_4_, the liver recovered with reduced fibrosis (Fig. 8b). Whole-mount fluorescence view of liver and immunostaining on liver sections showed that Mfsd2a-derived hepatocytes occupied both PP and PC regions during recovery (Fig. 8c,d). *In situ* hybridization showed increased expression of PP genes including *Arg1*, *Gls2* and *Pck1* in liver sections of CCl_4_-induced mice compared with that of oil-treated controls (Fig. 8e). Expression of PC metabolic genes including *Rnase4*, *Cyp2e1*, *Cyp1a2*, *Gs* and *Oat* were significantly reduced during injury compared with oil-treated controls and returned to near-normal level at 2 weeks after removal of CCl_4_ (Fig. 8e), indicating the metabolic reprogramming of Mfsd2a-derived PP hepatocytes into the PC hepatocytes. Moreover, expression of the PP gene E-cadherin (*CDH1*) was restricted to Mfsd2a-derived PP hepatocytes in livers of the oil-treated control mice. When PP hepatocytes expanded into the PC regions, CDH1 expression was also detected in RFP^+^ hepatocytes of the PC regions (Supplementary Fig. 13). However, 2 weeks after removal of CCl_4_, the PP gene marker CDH1 was no longer expressed by RFP^+^ hepatocytes of the PC regions, indicating reprogramming of these Mfsd2a-derived hepatocytes into a CDH1-negative cell fate in the PC region during recovery. Taken together, our results suggested that Mfsd2a-derived hepatocytes adopted a PC cell fate with re-establishment of metabolic zonation during liver regeneration.

**Figure 9:**
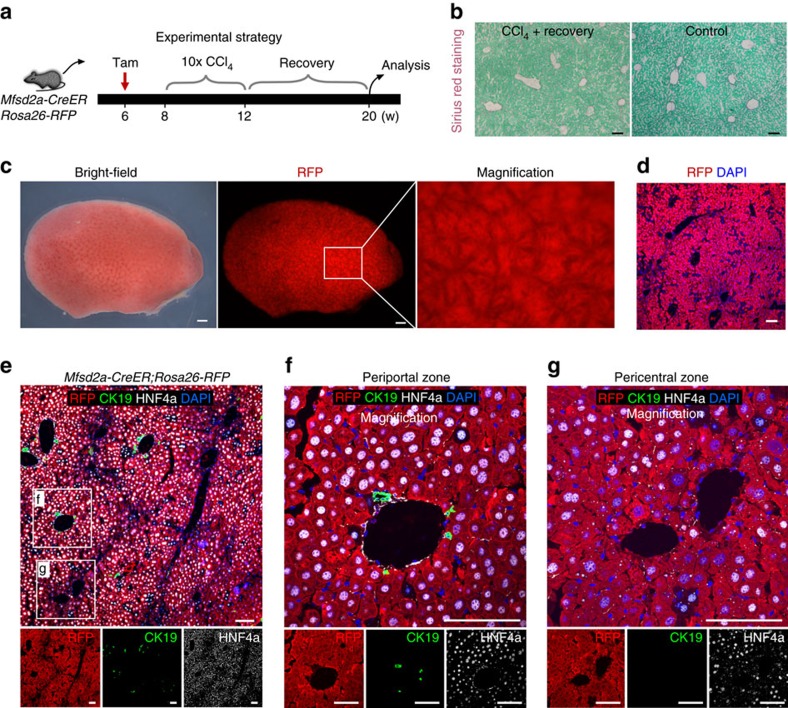
Mfsd2a-derived hepatocytes remain throughout the entire liver lobule after recovery from liver injury. (**a**) Schematic figure showing strategy for tamoxifen induction (Tam), CCl_4_ treatment and analysis after recovery. (**b**) Sirius red staining on liver sections collected from CCl_4_-treated mice after recovery and control mice (no CCl_4_ treatment). Scale bars, 100 μm. (**c**) Whole-mount bright-field and fluorescence view of liver after recovery from CCl_4_ treatment. Scale bars, 1 mm. (**d**) Immunostaining for RFP on liver sections showing RFP^+^ cells retained throughout entire liver lobules after recovery. Scale bars, 100 μm. (**e**–**g**) Immunostaining for RFP, CK19 and HNF4a on liver sections showing most hepatocytes in PP zone (**f**) and PC zone (**g**) as RFP^+^; **f**,**g** are magnified images of boxed regions in **e**. Scale bars, 100 μm. Each image is a representative of four individual samples.

We then asked whether these RFP^+^ hepatocytes in the PC region receded after long-term recovery. We therefore extended the recovery period for *Mfsd2a-CreER;Rosa26-RFP* mice to 20 weeks post chronic CCl_4_-induced injury (Fig. 9a). Our results of Sirius red staining showed that there was no significant difference in the degree of fibrosis between the recovery group and oil-treated control group (Fig. 9b). Whole-mount bright-field and fluorescence views of liver showed that the entire liver was RFP^+^ at 20 weeks old (Fig. 9c), which was confirmed by sectional immunostaining for RFP (Fig. 9d). Immunostaining for RFP, CK19 and HNF4a also showed that almost all HNF4a^+^ hepatocytes were RFP^+^ in both the PP and PC regions (Fig. 9e–g). Together, our data demonstrated that there was no survival privilege of PC hepatocytes (Mfsd2a negative) after chronic injury, whereas the PP hepatocytes expanded to completely repopulate the liver lobule throughout the entire liver.

### Reduced Mfsd2a^+^ PP hepatocytes are found during cholestatic injury

**Figure 10:**
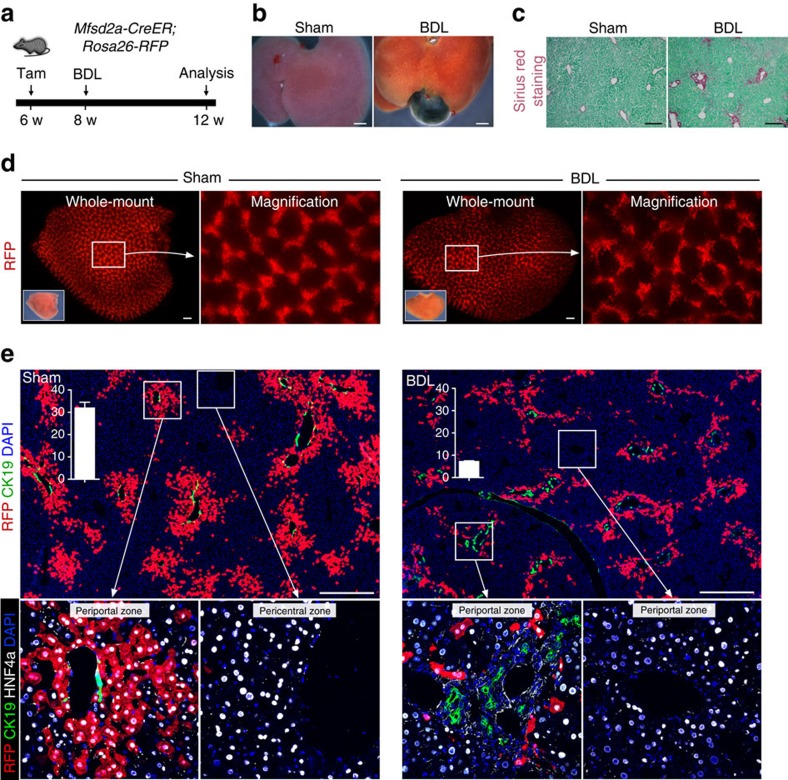
Reduction of pre-labelled PP hepatocytes after injury induced by BDL. (**a**) Schematic figure showing strategies for PP hepatocytes labelling and injury model induced by BDL. (**b**) Whole-mount bright view of sham and BDL livers. Scale bars, 2 mm. (**c**) Sirius red staining of liver sections. Scale bars, 200 μm. (**d**) Whole-mount fluorescence view of sham and BDL livers. Inserts are bright-field images of the same liver. Scale bars, 500 μm. (**e**) Immunostaining for RFP, CK19 and HNF4a on sham or BDL liver sections. Boxed regions are magnified in lower panels. Insertions indicate the quantification of the percentage of RFP^+^ hepatocytes in sham or BDL livers. *n*=4. Error bars are s.e.m. of the mean. Scale bars, 500 μm. Each image is a representative of four individual samples.

In addition to the two-third PH and chronic liver injury models, we also employed the biliary injury model via bile duct ligation (BDL), to study the cellular dynamics of hepatocyte repopulation during cholestatic injures (Fig. 10a). Whole-mount view of liver and Sirius red staining of liver sections confirmed that BDL injury led to severe liver jaundice and excessive fibrosis (Fig. 10b,c). Immunostaining for the ductal cell markers CK19, EPCAM and A6 showed strong ductal reaction in BDL injury (Supplementary Fig. 14). We then induced labelling of Mfsd2a^+^ PP hepatocytes 2 weeks before injury by tamoxifen injection into *Mfsd2a-CreER;Rosa26-RFP* mice. Lineage tracing of Mfsd2a^+^ PP hepatocytes in cholestatic liver showed a significant reduction in pre-labelled PP hepatocytes after BDL injury, with only two or three layers of RFP^+^ hepatocytes remaining in the PP regions compared with that of the sham-operated littermate controls within the same time window (%RFP^+^ hepatocytes: 31.89±2.62% versus 7.14±0.35%, respectively, *n*=4; Fig. 10d,e). It is likely to be that PP hepatocytes were prone to injury induced by bile duct obstruction and replaced by non-labelled hepatocytes such as PC hepatocytes during cholestasis.

We also used the 3,5-diethoxycarbonyl-1,4-dihydrocollidine (DDC)-containing diet to induce cholestatic injury model (Supplementary Fig. 15a). We found that after chronic DDC treatment, RFP^+^ hepatocytes still remained in the PP but their number reduced as sparsely distributed RFP^+^ cells were found in the PP region (Supplementary Fig. 15b). Detailed examination of these RFP^+^ cells by *Z*-stack confocal microcopy showed that a subset of RFP^+^ cells expressed CK19 but not HNF4a and adopted biliary epithelial cell shape and morphology (Supplementary Fig. 15c). Therefore, our data suggested that Mfsd2a^+^ hepatocytes regressed during cholestatic injury and may switch their cell fate during chronic injury.

Finally, we attempted to examine Mfsd2a expression by hepatocytes in all injury models of the present study including BDL, PH, CCl_4_ and DDC. Immunostaining for ESR and RFP showed that Mfsd2a expression was mainly restricted in the PP region post PH, CCl_4_ and DDC injuries. On the other hand, Mfsd2a expression was enriched in the PC region post BDL-induced injury (Supplementary Fig. 16). Nevertheless, detailed mechanisms for the distinct Mfsd2a expression by hepatocytes following different models of injury merit further investigations.

## Discussion

Self-renewal of hepatocytes within hepatic zonation has been regarded as the main mechanism by which liver regenerates. The present study revealed that hepatocytes were heterogeneous with repopulation priority of one population over another during liver regeneration. We provided evidence that the PP hepatocytes were more involved in self-expansion in response to regenerative cues compared with the PC hepatocytes post injury. Using the chronic injury model, we demonstrated that the Mfsd2a^+^ hepatocytes were the major source responsible for liver regeneration, as almost all hepatocytes were labelled as RFP^+^ during injury in the *Mfsd2a-CreER;Rosa26-RFP* lineage tracing line. The evidence of organ-wide cellular compensation by PP hepatocytes post liver injury could be of relevance to liver tumorigenesis after chronic liver injury^18^.

Under normal condition, the hepatocyte turnover rate is very slow during homeostasis^19^. After injury, the liver is capable of completely restoring its mass within a few weeks^20^. The cell replacement or compensatory growth in liver after injury could be achieved by stem cell differentiation or replication of pre-existing differentiated hepatocytes. Although there is an ongoing debate on whether there are facultative stem cells for hepatocyte regeneration, it becomes increasingly clear that replication of pre-existing hepatocytes appears to be the main mechanism by which liver regenerates as revealed in multiple injury and regeneration models^21^. However, hepatocytes are not uniform in terms of their molecular expression profiles and functions, and they are classified into different subpopulations based on their physical location in the liver lobule^7^,^8^. Our work identified Mfsd2a as a specific marker for hepatocytes in the PP region. Using genetic lineage tracing strategy, we found that Mfsd2a^+^ PP hepatocytes reduced in number during liver homeostasis. Font-Burgada *et al*.^13^ has previously reported that Sox9^+^ hybrid hepatocytes are a subpopulation of PP hepatocytes regenerated during injury without giving rise to liver cancer. Nevertheless, the Sox9-CreER line used in their study labels only one cell layer of hepatocytes surrounding the portal vein, in addition to biliary epithelial cells^13^. Our Mfsd2a-CreER labelled a broader region than Sox9-CreER, suggesting that Mfsd2a-CreER might not precisely target the same specific layer of hepatocytes. However, our Mfsd2a-CreER line used in this study labelled multiple layers of hepatocytes surrounding the portal vein, which were more properly defined as the PP zonation (Supplementary Fig. 1)^11^. Nevertheless, each line has unique advantages in addressing different questions. Therefore, the combinatory use of both Sox9- and Mfsd2a-based recombinases in the future could be valuable in understanding functions of various sub-populations of PP hepatocytes. Based on our extensive cell-labelling strategies for PP hepatocytes, we identified the gradual reduction of Mfsd2a-expressing PP hepatocytes during homeostasis. In fact, our findings are consistent with a recent work, which shows that Axin2^+^ hepatocytes reside in close proximity to the PC vein and expand to replace as much as 30% of hepatocytes of the entire liver after 1 year tracing^14^. The source of expanding Mfsd2a-negative hepatocytes in our study was likely to be the Axin2^+^ PC hepatocytes. Purification and characterization of Mfsd2a^+^ and Mfsd2a^−^ hepatocytes confirmed additional new PP or PC zonation markers. Therefore, it would be worthwhile to generate new genetic tools based on these markers to specifically label PC hepatocytes in future studies, which would provide better understandings in the cellular dynamics of PC hepatocytes during liver homeostasis.

It is important to note that the *Mfsd2a-CreER* line used in our study is ‘leaky', as we observed a few hepatocytes as RFP^+^ in the *Mfsd2-CreER;Rosa26-RFP* mice even without tamoxifen induction. These RFP^+^ hepatocytes exhibited similar proliferation property compared with the unlabelled PP hepatocytes found at day 2 after CCl_4_ injection. Nevertheless, these ‘leaky' hepatocytes only contributed to a small amount of hepatocytes in the entire liver when compared with the complete replacement of hepatic zonation by RFP^+^ hepatocytes in the tamoxifen-treated livers after CCl_4_ treatment (Supplementary Fig. 9d,e). Therefore, we could still conclude that the PP Mfsd2a^+^ hepatocytes replaced PC hepatocytes and repopulated the entire liver after CCl_4_-induced chronic injury.

In this study, we also observed that the Mfsd2a^+^ hepatocytes responded differently in various liver injury models including hepatectomy, CCl_4_-induced hepatotoxicity, BDL-induced cholestasis and DDC-induced injury. Our results indicated that varied injuries could stimulate diverse signal pathways that triggered cell proliferation or cell death among Mfsd2a^+^ or Mfsd2a^−^ hepatocyte populations. For instance, it is known that hepatocytes in the two metabolic zonations express different metabolic enzymes that are responsible for digestion and toxicity removal^8^. CCl_4_-induced hepatotoxicity is caused by free radicals, which are metabolic products of CCl_4_ mainly formed by Cyp2e1. Our *in situ* hybridization data showed that Cyp2e1 was highly expressed in the PC region, which accounts for significant toxicity of hepatocytes in the PC zonation after CCl_4_ treatment. Similar to many PC genes including glutamate synthetase (GS), Cyp2e1 is also regulated by the Wnt signalling pathway in the PC region where β-catenin is activated^7^. On the contrary, the PP genes such as *Arg1*, *Gls2* and *Pck1* are not tightly regulated by Wnt signalling pathway in the PP region, where β-catenin-negative regulator Apc is highly expressed^8^. Our study revealed that Mfsd2a^+^ PP hepatocytes could expand as a compensatory growth response to completely occupy the PC region. We found that these Mfsd2a-derived hepatocytes gradually lost PP gene markers and regained PC cell fate with expression of PC-specific gene markers such as Rnase4, Cyp2e1, Cyp1a2, GS and Oat after recovery. Therefore, our results demonstrated that hepatocytes, at a population level, could be metabolically reprogrammed and replaced entirely over one another. This metabolic reprogramming or cell-fate change with respective genetic alternation is likely to be attributed to the hepatic niches in the PC veins where endothelial cells secrete Wnt ligands Wnt2 and Wnt9b^14^. Whether the expansion and reprogramming of pre-existing PP hepatocytes during liver regeneration depend on Wnt and other signalling pathways^11^,^12^,^14^, as well as the cues from the local microenvironment such as blood flow, remain unanswered and require further investigations.

## Methods

### Mice

All animal studies were carried out in accordance with the guidelines of the Institutional Animal Care and Use Committee of the Institute for Nutritional Sciences, Shanghai Institutes for Biological Sciences, Chinese Academy of Science. Mice were maintained on a C129/C57BL6/J;ICR mixed background. *Mfsd2a-CreER* mouse line was generated by homologous recombination using the CRISPR/Cas9 technology. A complementary DNA encoding Cre recombinase fused with a mutant form of the ESR hormone-binding domain (CreER^T2^)^22^ was inserted in frame with the translational start codon of the *Mfsd2a* gene. The *Mfsd2a-CreER* line was generated by Shanghai Biomodel Organism Co., Ltd and Rosa26-RFP was kindly provided by Hongkui Zeng at Allan Institute^23^. Tamoxifen (Sigma, T5648) was dissolved in corn oil and administered to mice at the indicated time. Adult male mice received 0.1–0.2 mg tamoxifen per gram mouse body weight by oral gavage and neonatal mice received intraperitoneal injections of tamoxifen^24^.

### Genomic PCR

Genomic DNA was prepared from embryonic yolk sac or mouse tail. Tissues were lysed by incubation with Proteinase K overnight at 55 °C, followed by centrifugation at maximum speed for 5 min, to obtain supernatant with genomic DNA. DNA was precipitated by adding isopropanol and washed in 70% ethanol. All embryos or mice were genotyped with specific primers that distinguish knock-in allele from wild-type allele. For *Rosa26-RFP* line, primers 5′-GGCATTAAAGCAGCGTATCC-3′ and 5′-CTGTTCCTGTACGGCATGG-3′ were used to detect the RFP-positive allele. For *Mfsd2a-CreER* line, primers 5′-TGATGGGGATTCAGAAGTCAAGG-3′ and 5′-GGACAGAAGCATTTTCCAGGTATG-3′ were used to detect the Cre-positive allele and 5′-CCCCTTTCCTGGGTTGTAAAATG-3′ and 5′-GGGCTTCAGACTTTCTCCATCAC-3′ were used to detect the wild-type allele.

### Injury model

Two-third PH was performed to induce injury model^25^. In detail, 8-week-old mice were anaesthetized with 2% isoflurane and oxygen flow in a plexiglas chamber. The abdominal fur was removed and the skin was disinfected. The mice were transferred onto a 37 °C heat pad and maintained anaesthesia by inhalation of isoflurane with oxygen. A midline abdominal skin and muscle incision was made to expose the liver. The left lobe and the median lobe were tied and cut. The peritoneum was closed with a 5–0 suture and skin was closed afterwards. After closing the abdomen, the skin surrounding the suture was disinfected with betadine and the animal was placed on a warming pad for recovery. For the CCl_4_-induced acute injury, mice were intraperitoneally injected a single does of 1 μl per gram body weight CCl_4_ (ref. 5). For the CCl_4_-induced chronic injury model, CCl_4_ was dissolved at 1:3 in corn oil and injected intraperitoneally at a dose of 1 μl per gram body weight every 3 days for ten times^5^. BDL injury model was carried out according to established protocols^26^. Anaesthesia procedure was the same as PH. A midline abdominal incision was made to expose the liver. The common bile duct was ligated twice with 4–0 silk sutures. Sham-operated animals served as controls. For DDC feeding, mice received mouse diet (Harlan Teklad, 5015) containing 0.1% DDC (Sigma-Aldrich).

### Immunostaining

Immunostaining was performed according to standard protocols^27^. In detail, tissues were dissected in PBS and fixed in 4% paraformaldehyde (PFAl Sigma) at 4 °C for 1 h. Afterwards, tissues were washed in PBS for three times, dehydrated in 30% sucrose overnight at 4 °C and embedded in OCT (Sakura). Ten-micrometre cryosections were obtained and air-dried afterwards at room temperature. For staining, dried sections were washed in PBS and then blocked with 5% normal donkey serum (Jackson Immunoresearch) and 0.1% Triton X-100 in PBS for 30 min at room temperature. Sections were incubated with the primary antibodies overnight at 4 °C. The following antibodies were used: RFP (Rockland, 600-401-379, 1:200), cytokeratin 19 (Developmental Studies Hybridoma Bank, TROMA-III, 1:100), HNF4a (Santa Cruz, sc-6556, 1:100), CD31 (BD Pharmingen, 553370, 1:1,000), VE-cadherin (R & D, AF1002, 1:100), ESR (Abcam, ab27595, 1:100), Desmin (R&D, AF3844, 1:100), PDGFRa (R & D, AF1062, 1:100), EPCAM (Abcam, ab92383, 1:100), SOX9 (Millipore, AB5535, 1:100), aSMA (Sigma, F3777, 1:100), E-cadherin (Cell Signaling, 3195, 1:100), Ki67 (lab vision, RM-9106-F1, 1:100), COL3A1 (Southernbiotech, 1330-01, 1:500) and A6 (a gift from Valentian Factor, 1:100). Signals were developed with Alexa fluorescence antibodies (Invitrogen) and nuclei were stained with 4,6-diamidino-2-phenylindole (Vector labs). Horseradish peroxidase-conjugated antibodies with tyramide signal amplification kit (Perkin Elmer) was used to amplify weak signals. Before coverslip addition, tissues were counterstained with 4,6-diamidino-2-phenylindole (Vector lab). Immunostaining images were acquired by Olympus fluorescence microscope (BX53), Zeiss stereomicroscope (AXIO Zoom, V16), Zeiss confocal laser scanning microscope (LSM510) and Olympus confocal microscope (FV1200).

### Hepatocyte proliferation assay

Mice aged 8–10 weeks received intraperitoneal injection of 50 mg kg^−1^ EdU (Invitrogen, A10044) on 7 consecutive days and were killed 6 h after the last EdU dose. Cryosections were obtained and incubated with the primary and secondary antibodies, and EdU signal was detected using the Click-iT EDU Alexa Fluor 488 Imaging Kit (Life Technologies, C10337) following the manufacturer's instructions. Images were acquired by Olympus confocal microscope (FV1200).

### Sirius red staining

Sirius red staining was aimed to assess fibrotic tissue formation after chronic injury^28^. In detail, cryosections were fixed in 4% PFA for 15 min, then washed in PBS for 15 min and fixed overnight in Bouins solution (5% acetic acid, 9% formaldehyde and 0.9% picric acid). Subsequently, sections were stained with 0.1% Fast Green (Fisher) for 3 min and incubated in 1% acetic acid for 1 min followed by incubation with 0.1% Sirius red (Sigma) for 2 min. Sections were rinsed with tap water before incubation into the staining solution. Slides were dehydrated in 100% ethanol twice, cleared in xylene and mounted with resinous medium. Images were obtained on an Olympus microscope (BX53).

### *In situ* hybridization

*In situ* hybridization was performed according to published protocols^29^. Liver tissues were fixed in 4% PFA overnight and dehydrated in 30% sucrose followed by embedding in OCT (Sakura). All of the reagents were dissolved in diethy pyrocarbonate-treated PBS. Cryosections (10 μm) were hybridized with digoxigenin-labelled antisense probe (1 μg mg^−1^) at 65 °C overnight. After washing in maleic acid buffer containing Tween 20 (MABT) buffer and 1 × SSC buffer, slides were incubated with blocking buffer (10% sheep serum and 2% blocking reagent in maleic acid buffer containing Tween 20 buffer) and subsequently incubated with anti-digoxigenin antibody (Roche, 11093274910) diluted in blocking buffer at 4 °C overnight. Signal was developed with BCIP/NBT (Promega, S3771) in dark. Images were acquired on an Olympus microscope (BX53). The primers are listed in Supplementary Table 1.

### FACS of hepatocytes

For gene expression analysis, we isolated RFP^+^ and RFP^−^ hepatocytes from the liver of *Mfsd2a-CreER;Rosa26-RFP* mice. Hepatocytes were first isolated by two-step collagenase perfusion technique^30^. In detail, mice were anaesthetized first and the dorsal side was placed down onto a tray when the mouse was no longer responsive. ‘U'-shaped incision was made through the skin and muscle of the lower abdomen to expose the liver with inferior vena cava and portal vein. A cannula was inserted into the inferior vena cava and secured with a suture around the cannula. Portal vein was cut and the mouse liver was perfused with perfusion medium I for 10–15 min. The liver was next perfused with medium containing collagenase type I (Invitrogen) for ∼10 min. After adequate digestion, the liver was dissected in petri dish and tissues were passed through a 70 μm filter followed by centrifugation for 2 min at 50 *g*. Non-parenchymal cells remained in the supernatant were discarded and hepatocytes pellet was resuspended in DMEM medium. The above washing step was repeated for three times. Washed cells from previous step were further purified by Percoll. Dissociated hepatocytes were purified by a FACS Aria II Flow Cytometer (BD Bioscience). Hepatocytes were separated by FACS based on size/granularity (FSC/SSC). Doublets were excluded by FSC-H and FSC-A analysis. RFP-positive or -negative hepatocytes were purified based on RFP expression.

### Total RNA extraction and quantitative RT–PCR

RNA extraction and quantitative RT–PCR were performed according to the manufacturer's protocols^31^. Sorted cells were centrifuged and lysed with Trizol. Total RNA was extracted according to the manufacturer's instructions (Invitrogen) and converted to cDNA using Prime Script RT kit (Takara). The SYBR Green qPCR master mix (Applied Biosystems) was used and quantitative RT–PCR was performed on ABI 7500 Real-Time PCR System (Applied Biosystems). A list of PCR primers was included in Supplementary Table 2.

### Statistics

Data for two groups were analysed by a two-tailed unpaired Student's *t*-test, whereas comparison between more than two groups was performed using an analsysi of variance followed by Tukey's multiple comparison test. Significance was accepted when *P*<0.05. All data were presented as mean±s.e.m.

### Data availability

Data supporting the findings of this study are available within the article and its Supplementary Information files, and from the corresponding author upon reasonable request.

## Additional information

**How to cite this article:** Pu, W. *et al*. Mfsd2a^+^ hepatocytes repopulate the liver during injury and regeneration. *Nat. Commun.* **7,** 13369 doi: 10.1038/ncomms13369 (2016).

**Publisher's note:** Springer Nature remains neutral with regard to jurisdictional claims in published maps and institutional affiliations.

## Supplementary Material

Supplementary Information Supplementary Figures 1-16 and Supplementary Tables 1-2.

## References

[b1] Yanger K. . Robust cellular reprogramming occurs spontaneously during liver regeneration. Genes Dev. 27, 719–724 (2013). 23520387 10.1101/gad.207803.112 PMC3639413

[b2] Tarlow B. D. . Bipotential adult liver progenitors are derived from chronically injured mature hepatocytes. Cell Stem Cell 15, 605–618 (2014). 25312494 10.1016/j.stem.2014.09.008 PMC4254170

[b3] Grompe M. Liver stem cells, where art thou? Cell Stem Cell 15, 257–258 (2014). 25192457 10.1016/j.stem.2014.08.004

[b4] Miyajima A., Tanaka M. & Itoh T. Stem/progenitor cells in liver development, homeostasis, regeneration, and reprogramming. Cell Stem Cell 14, 561–574 (2014). 24792114 10.1016/j.stem.2014.04.010

[b5] Malato Y. . Fate tracing of mature hepatocytes in mouse liver homeostasis and regeneration. J. Clin. Invest. 121, 4850–4860 (2011). 22105172 10.1172/JCI59261 PMC3226005

[b6] Yanger K. . Adult hepatocytes are generated by self-duplication rather than stem cell differentiation. Cell Stem Cell 15, 340–349 (2014). 25130492 10.1016/j.stem.2014.06.003 PMC4505916

[b7] Burke Z. D. . Liver zonation occurs through a beta-catenin-dependent, c-Myc-independent mechanism. Gastroenterology 136, 2316–2324 e2311-2313 (2009). 19268669 10.1053/j.gastro.2009.02.063

[b8] Benhamouche S. . Apc tumor suppressor gene is the ‘zonation-keeper' of mouse liver. Dev. Cell 10, 759–770 (2006). 16740478 10.1016/j.devcel.2006.03.015

[b9] Stanger B. Z. Probing hepatocyte heterogeneity. Cell Res. 25, 1181–1182 (2015). 26403190 10.1038/cr.2015.117 PMC4650421

[b10] Jungermann K. & Katz N. Functional specialization of different hepatocyte populations. Physiol. Rev. 69, 708–764 (1989). 2664826 10.1152/physrev.1989.69.3.708

[b11] Colnot S. & Perret C. Liver zonation. Mol. Pathol. Library 5, 7–16 (2011).

[b12] Braeuning A. . Differential gene expression in periportal and perivenous mouse hepatocytes. FEBS J. 273, 5051–5061 (2006). 17054714 10.1111/j.1742-4658.2006.05503.x

[b13] Font-Burgada J. . Hybrid periportal hepatocytes regenerate the injured liver without giving rise to cancer. Cell 162, 766–779 (2015). 26276631 10.1016/j.cell.2015.07.026 PMC4545590

[b14] Wang B., Zhao L., Fish M., Logan C. Y. & Nusse R. Self-renewing diploid Axin2(+) cells fuel homeostatic renewal of the liver. Nature 524, 180–185 (2015). 26245375 10.1038/nature14863 PMC4589224

[b15] Nguyen L. N. . Mfsd2a is a transporter for the essential omega-3 fatty acid docosahexaenoic acid. Nature 509, 503–506 (2014). 24828044 10.1038/nature13241

[b16] Ben-Zvi A. . Mfsd2a is critical for the formation and function of the blood-brain barrier. Nature 509, 507–511 (2014). 24828040 10.1038/nature13324 PMC4134871

[b17] Planas-Paz L. . The RSPO-LGR4/5-ZNRF3/RNF43 module controls liver zonation and size. Nat. Cell Biol. 18, 467–479 (2016). 27088858 10.1038/ncb3337

[b18] Hailfinger S., Jaworski M., Braeuning A., Buchmann A. & Schwarz M. Zonal gene expression in murine liver: lessons from tumors. Hepatology 43, 407–414 (2006). 16496347 10.1002/hep.21082

[b19] Sell S. Heterogeneity and plasticity of hepatocyte lineage cells. Hepatology 33, 738–750 (2001). 11230756 10.1053/jhep.2001.21900

[b20] Michalopoulos G. K. & DeFrances M. C. Liver regeneration. Science 276, 60–66 (1997). 9082986 10.1126/science.276.5309.60

[b21] Kopp J. L., Grompe M. & Sander M. Stem cells versus plasticity in liver and pancreas regeneration. Nat. Cell Biol. 18, 238–245 (2016). 26911907 10.1038/ncb3309

[b22] Indra A. . Temporally-controlled site-specific mutagenesis in the basal layer of the epidermis: comparison of the recombinase activity of the tamoxifen-inducible Cre-ER(T) and Cre-ER(T2) recombinases. Nucleic Acids Res. 27, 4324–4327 (1999). 10536138 10.1093/nar/27.22.4324 PMC148712

[b23] Madisen L. . A robust and high-throughput Cre reporting and characterization system for the whole mouse brain. Nat. Neurosci. 13, 133–140 (2010). 20023653 10.1038/nn.2467 PMC2840225

[b24] Tian X. . Subepicardial endothelial cells invade the embryonic ventricle wall to form coronary arteries. Cell Res. 23, 1075–1090 (2013). 23797856 10.1038/cr.2013.83 PMC3760626

[b25] Mitchell C. & Willenbring H. A reproducible and well-tolerated method for 2/3 partial hepatectomy in mice. Nat. Protoc. 3, 1167–1170 (2008). 18600221 10.1038/nprot.2008.80

[b26] Tag C. G. . Bile duct ligation in mice: induction of inflammatory liver injury and fibrosis by obstructive cholestasis. J. Vis. Exp. 96, 52438–52440 (2015). 10.3791/52438 PMC4354634 25741630

[b27] Liu Q. . Genetic lineage tracing identifies in situ Kit-expressing cardiomyocytes. Cell Res. 26, 119–130 (2016). 26634606 10.1038/cr.2015.143 PMC4816131

[b28] He L. . Genetic lineage tracing discloses arteriogenesis as the main mechanism for collateral growth in the mouse heart. Cardiovasc Res. 109, 419–430 (2016). 26768261 10.1093/cvr/cvw005 PMC4752045

[b29] Zhang H. . Yap1 is required for endothelial to mesenchymal transition of the atrioventricular cushion. J. Biol. Chem. 289, 18681–18692 (2014). 24831012 10.1074/jbc.M114.554584 PMC4081914

[b30] Li W. C., Ralphs K. L. & Tosh D. Isolation and culture of adult mouse hepatocytes. Methods Mol. Biol. 633, 185–196 (2010). 20204628 10.1007/978-1-59745-019-5_13

[b31] Zhou B. . Adult mouse epicardium modulates myocardial injury by secreting paracrine factors. J. Clin. Invest. 121, 1894–1904 (2011). 21505261 10.1172/JCI45529 PMC3083761

