# Supplementary material for: Mfsd2a+ hepatocytes repopulate the liver during injury and regeneration
Source: Nat Commun. 2016 Nov 18;7:13369. doi: 10.1038/ncomms13369 (PMC5120209; doi:10.1038/ncomms13369)
Supplement: Supplementary Information — Supplementary Figures 1-16 and Supplementary Tables 1-2. [file ncomms13369-s1.pdf]

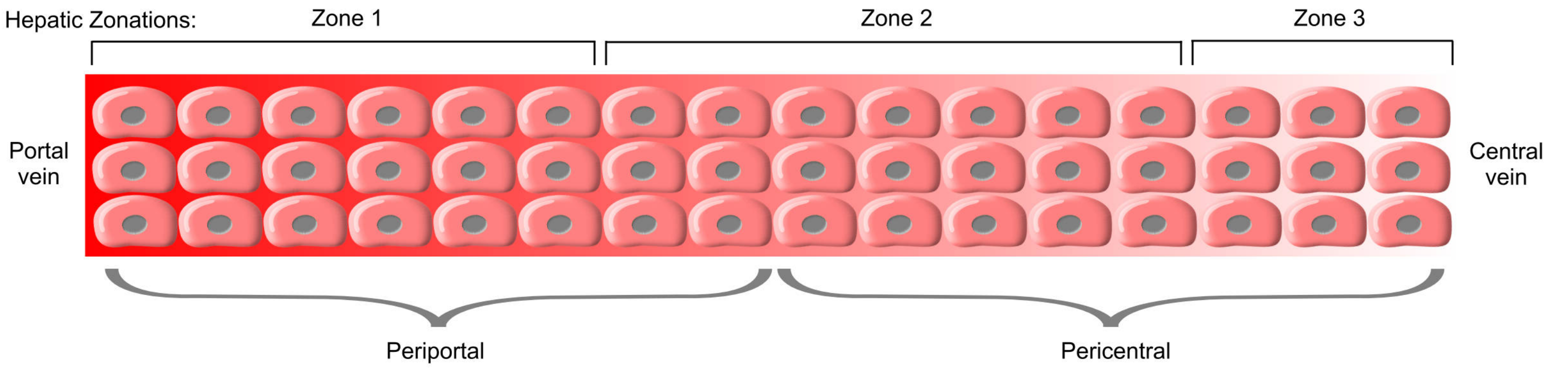

Supplementary Figure 1. Schematic figure showing periportal and pericentral regions covering different hepatic zonations. Periportal region consists of Zone 1 and partial of zone 2; pericentral region consists of Zone 3 and partial Zone 2. The numbers of hepatocytes allocated to Zone 1, 2, 3 are based on the book chapter of "Liver Zonation" from Reference (11).

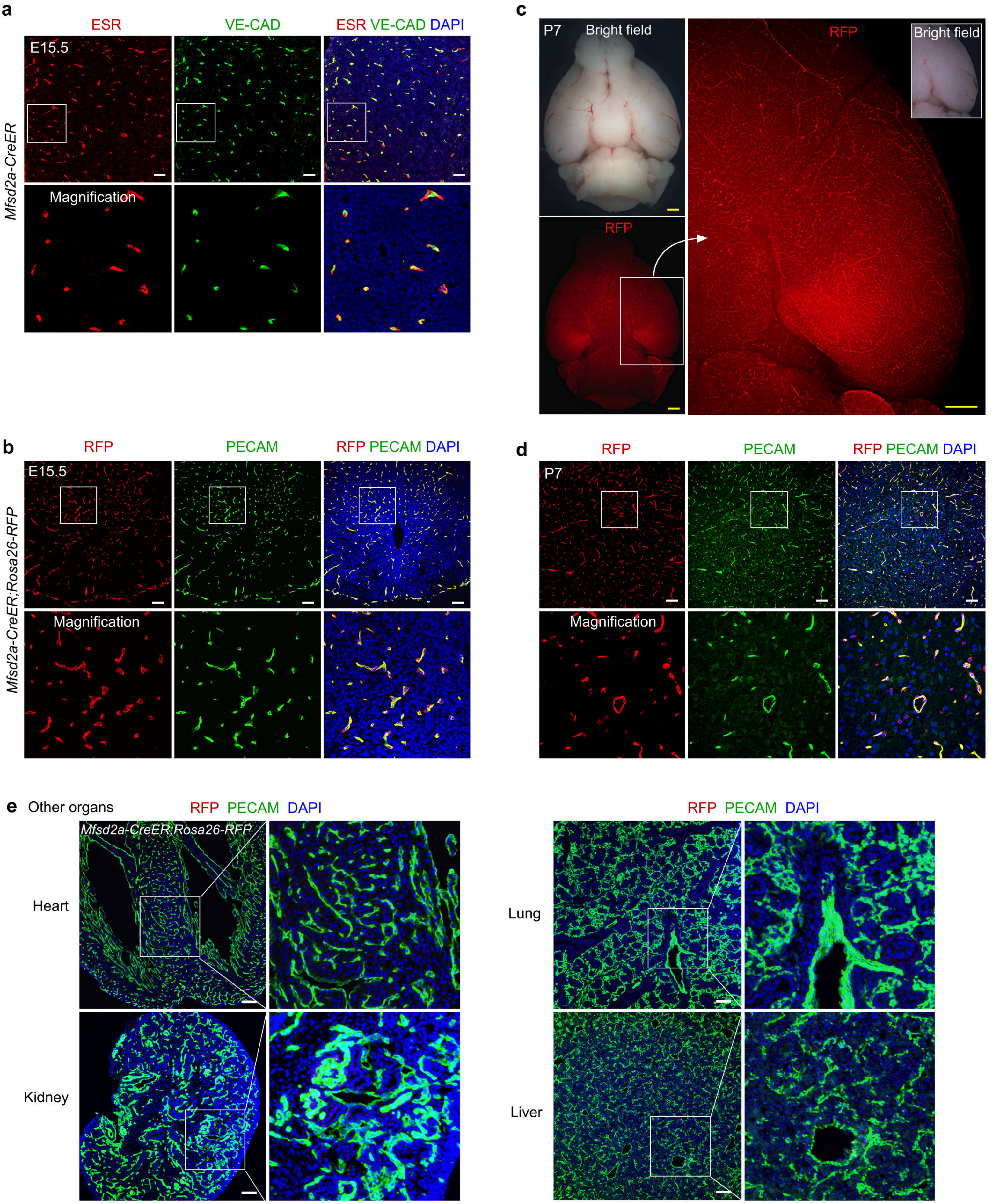

Supplementary Figure 2. *Mfsd2a-CreER* labels brain blood vessels. (**a**) Immunostaining for ESR and VE-CAD on E15.5 *Mfsd2a-CreER* mouse brain section. (**b**) Immunostaining for RFP and PECAM on the E15.5 *Mfsd2a-CreER;Rosa26-RFP* brain sections. Tamoxifen was administered at E13.5. (**c**) Whole mount bright and fluorescence view of brains from P7 *Mfsd2a-CreER;Rosa26-RFP* mice treated with tamoxifen two days before analysis. (**d**) Immunostaining for PECAM and RFP on brain sections of P7 *Mfsd2a-CreER;Rosa26-RFP* mice. (**e**) Immunostaining for RFP and PECAM on sections of other tissues. Tamoxifen was administered at E13.5 and tissues were collected at E15.5. Scale bars: yellow, 1 mm; white, 100 μm. Each image is a representative of 4 individual samples.

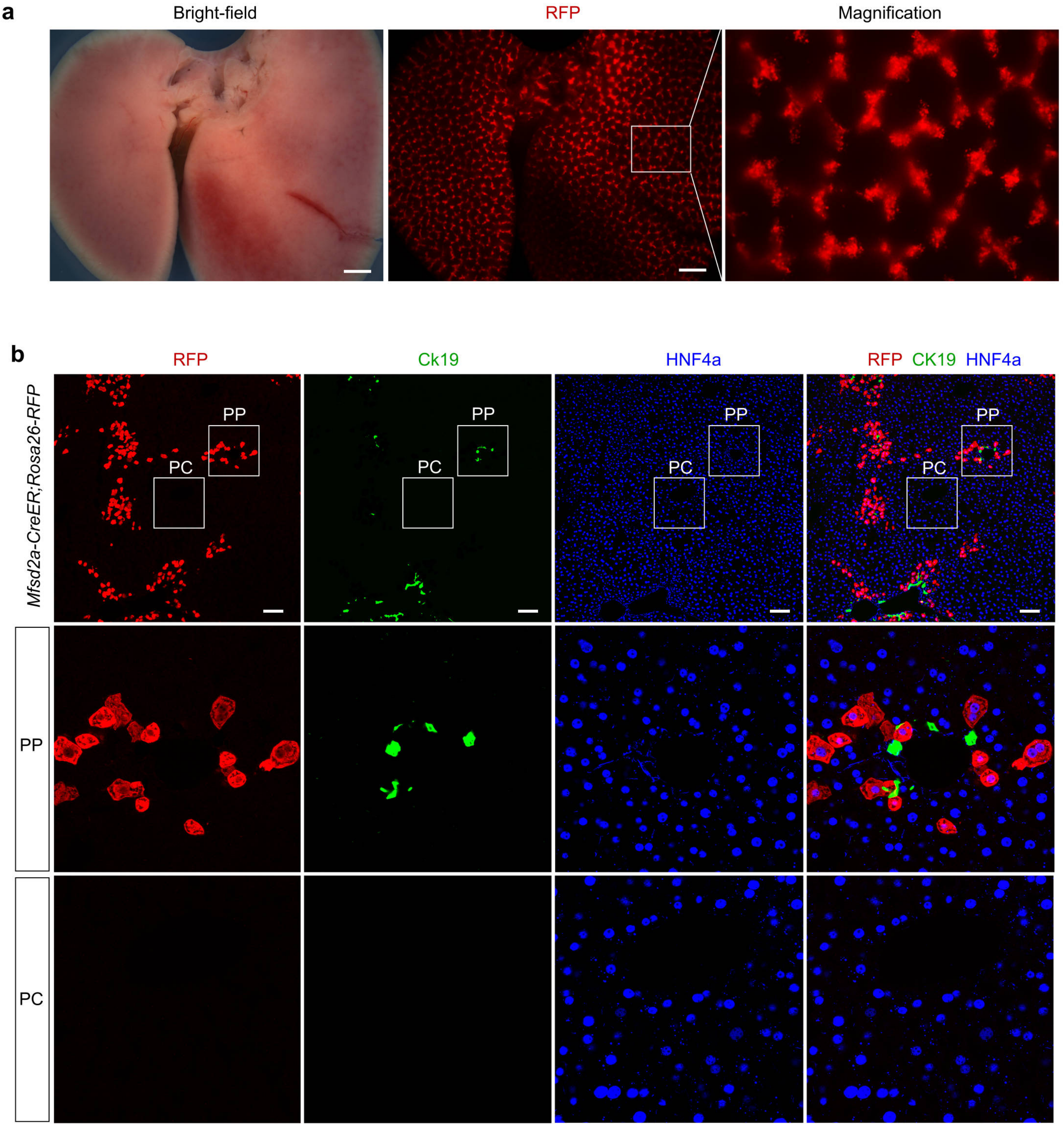

Supplementary Figure 3. Lineage tracing of Mfsd2a+ hepatocytes by low dose of tamoxifen treatment. (a) Whole-mount bright field and fluorescence view of *Mfsd2a-CreER;Rosa26-RFP* liver. (**b**) Immunostaining for RFP, CK19 and HNF4a on liver sections from *Mfsd2a-CreER;Rosa26-RFP* mice. Tamoxifen was injected at 6 weeks old. Periportal (PP) and pericentral (PC) regions are magnified in the lower panels. Scale bars, 1mm in a; 100 µm in b. Each image is a representative of 4 individual samples.

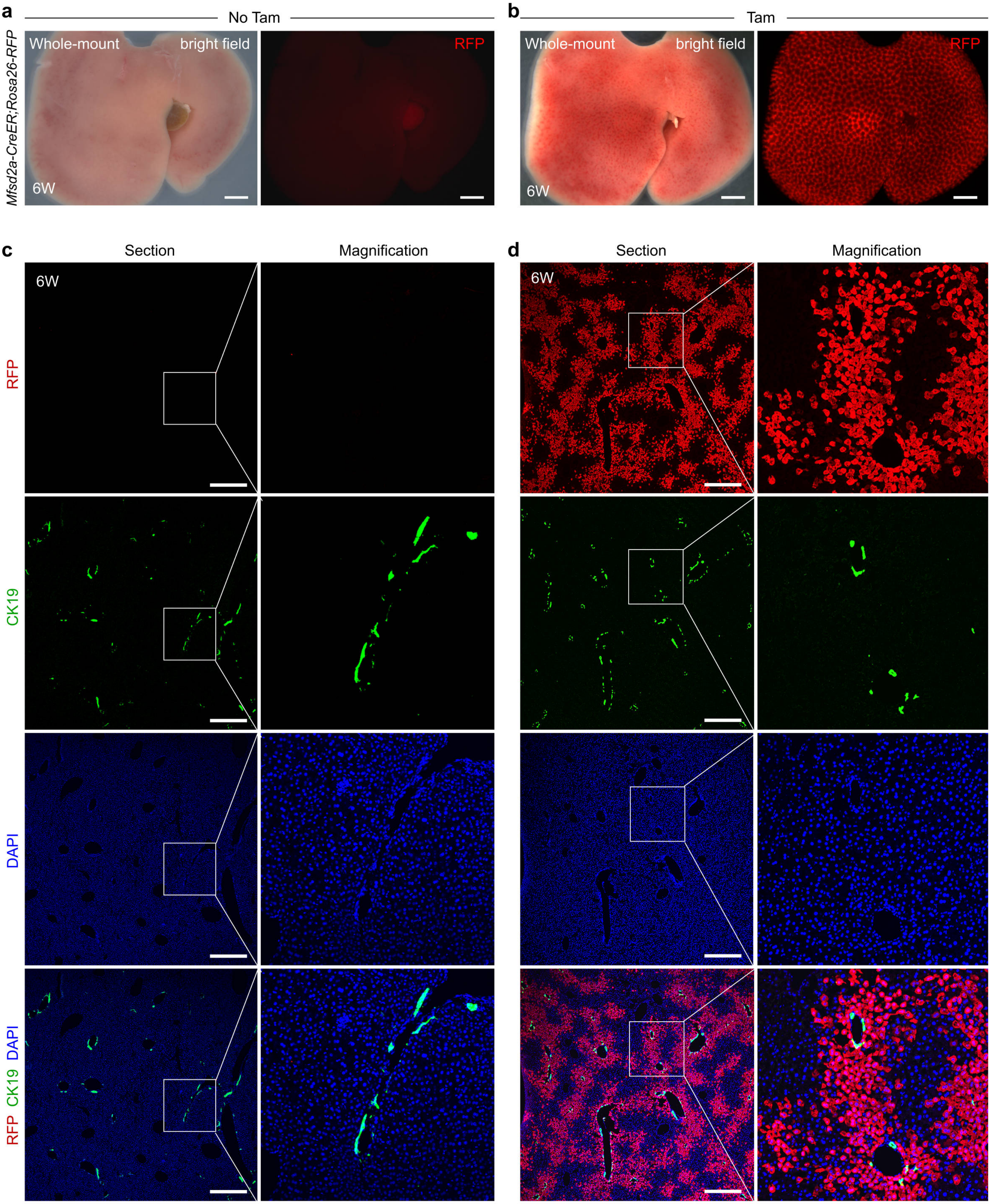

Supplementary Figure 4. Cell labeling by *Mfsd2a-CreER* in adult liver of mice treated with or without tamoxifen. (**a**,**b**) Whole-mount bright field or fluorescence view of livers from *Mfsd2a-CreER;Rosa26-RFP* mice without or with tamoxifen (Tam) administration. (**c**,**d**) Immunostaining for RFP and CK19 on liver sections. Scale bars, 2 mm in **a**,**b**; 500 μm in **c**,**d**. Each image is a representative of 4 individual samples.

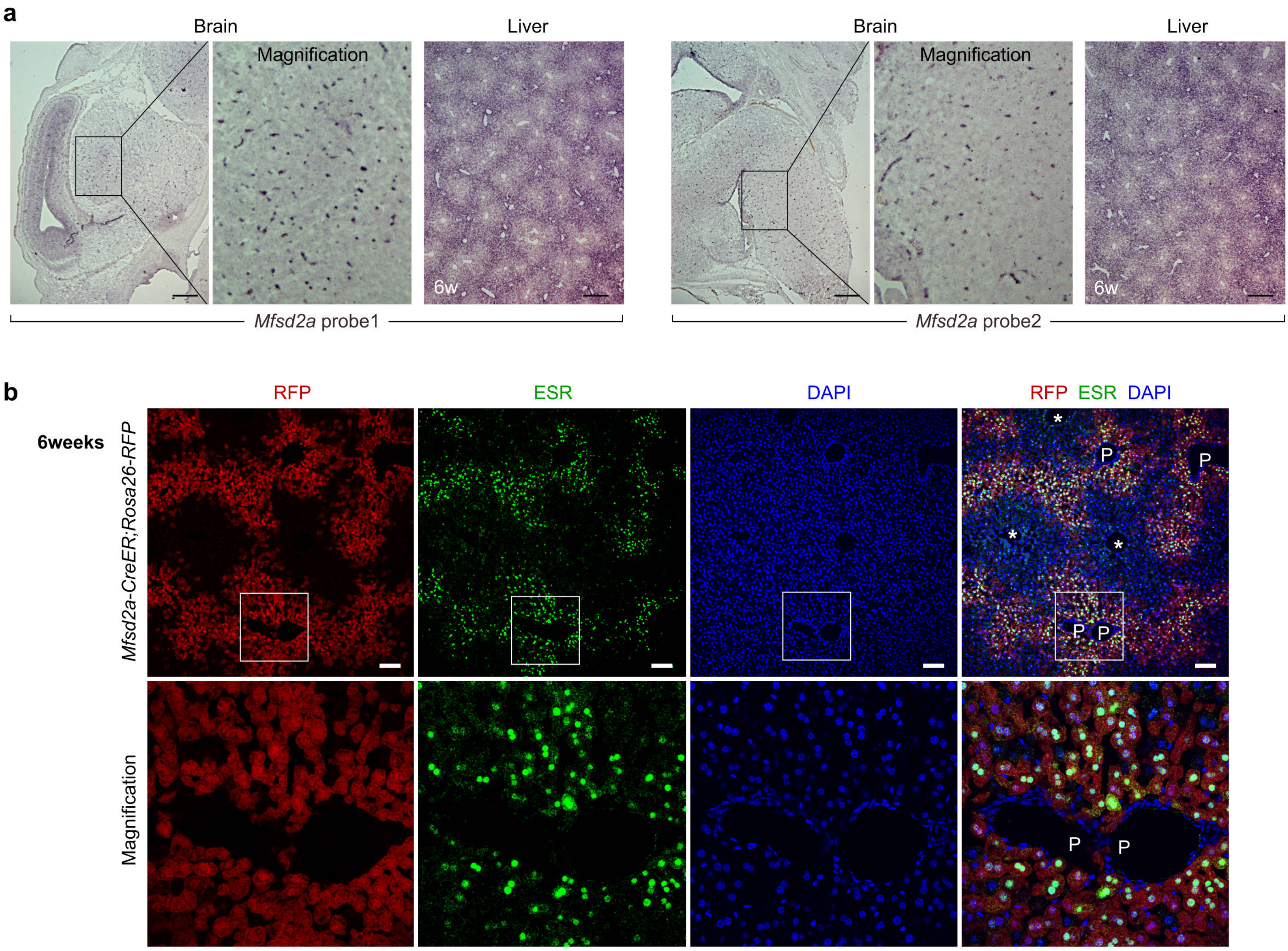

Supplementary Figure 5. Expression of Mfsd2a in brain and liver. (**a**) *In situ* hybridization of *Mfsd2a* on brain and liver sections. (**b**) Immunostaining for RFP and ESR on liver sections from 6 weeks old *Msd2a-CreER;Rosa26-RFP* mouse. Mouse tissue was collected within 48 hours of tamoxifen induction. ESR is expressed in hepatocytes close to portal vein (P) but not in hepatocytes close to central vein (*). Scale bars, 500 μm in a; 100 μm in b. Each image is a representative of 4 individual samples.

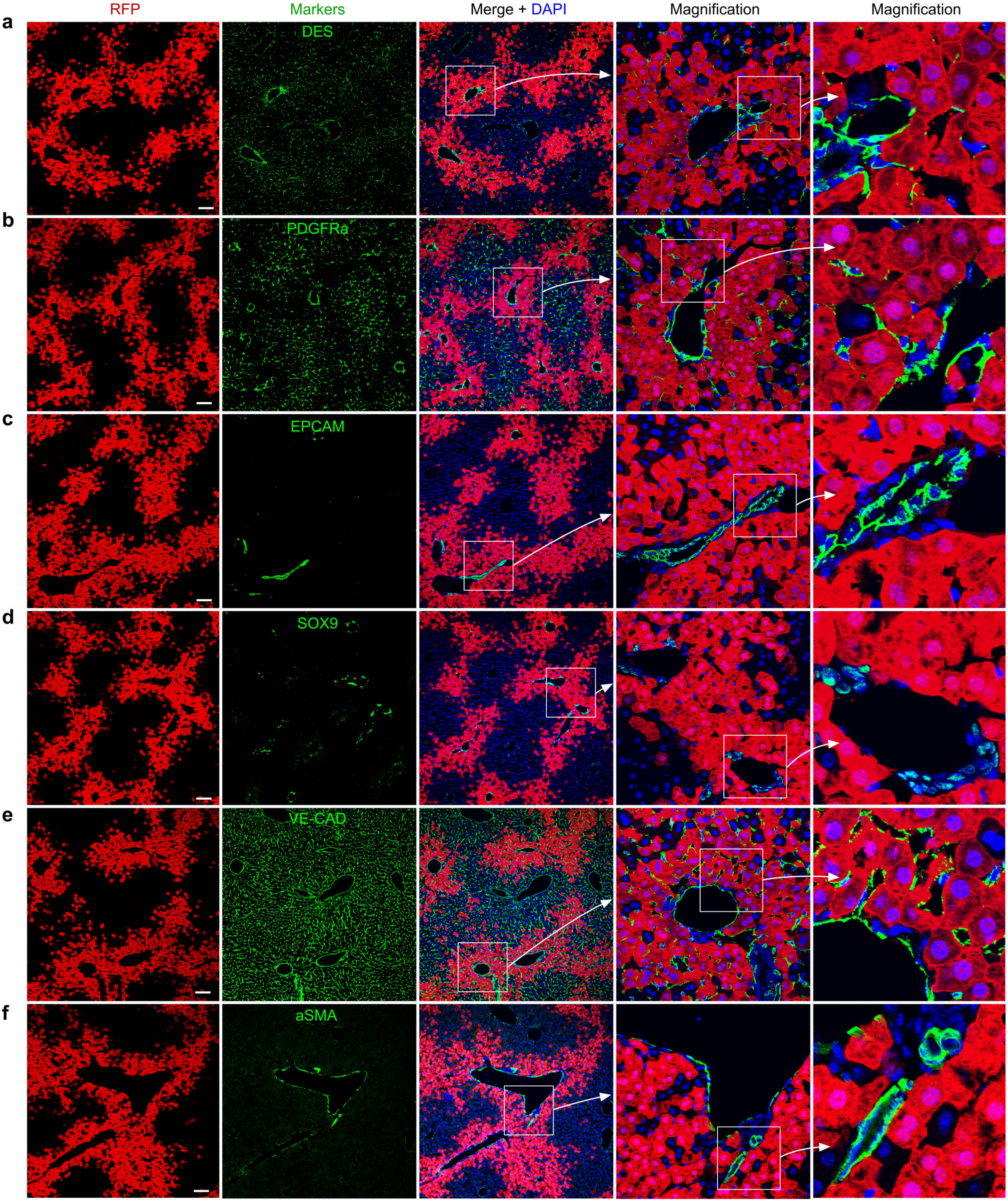

Supplementary Figure 6. *Mfsd2a-CreER* does not label non-hepatocyte. (**a**-**f**), Immunostaining for RFP and non-hepatocyte markers on *Mfsd2a-CreER;Rosa26-RFP* liver sections shows that RFP+ cells (red) are not DES+, PDGFRa+, EPCAM+, SOX9+, VE-CAD+, aSMA+ cells (green), suggesting *Mfsd2a-CreER* does not label stellate cells, fibroblasts, cholangiocytes, endothelial cells or smooth muscle cells in the adult liver. Nuclei were stained with DAPI (blue). Scale bars, 100 μm. Each image is a representative of 4 individual samples.

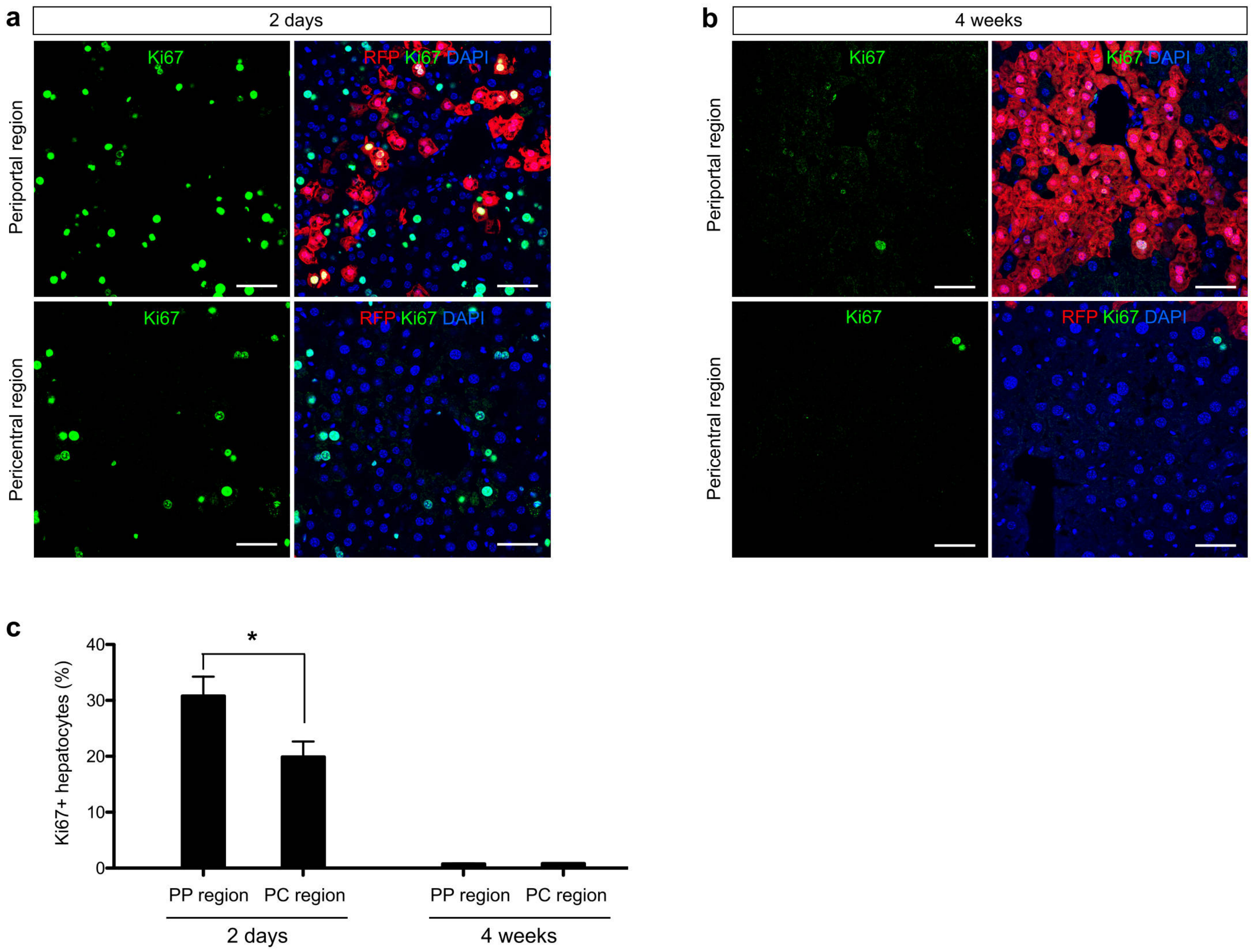

Supplementary Figure 7. Proliferation of hepatocytes in periportal and pericentral regions at early and late stages after partial hepatectomy. (**a**,**b**) Immunostaining for Ki67 and RFP on liver sections collected from *Mfsd2a-CreER;Rosa26-RFP* at day 2 and week 4 after partial hepatectomy (PH injury). Scale bars, 100 µm. (**c**) Quantification of percentage of Ki67+ hepatocytes in periportal (PP) or pericentral (PC) regions. *$P < 0.05$; n = 4 mice per group; two tailed unpaired *t*-test. Each image is a representative of 4 individual samples.

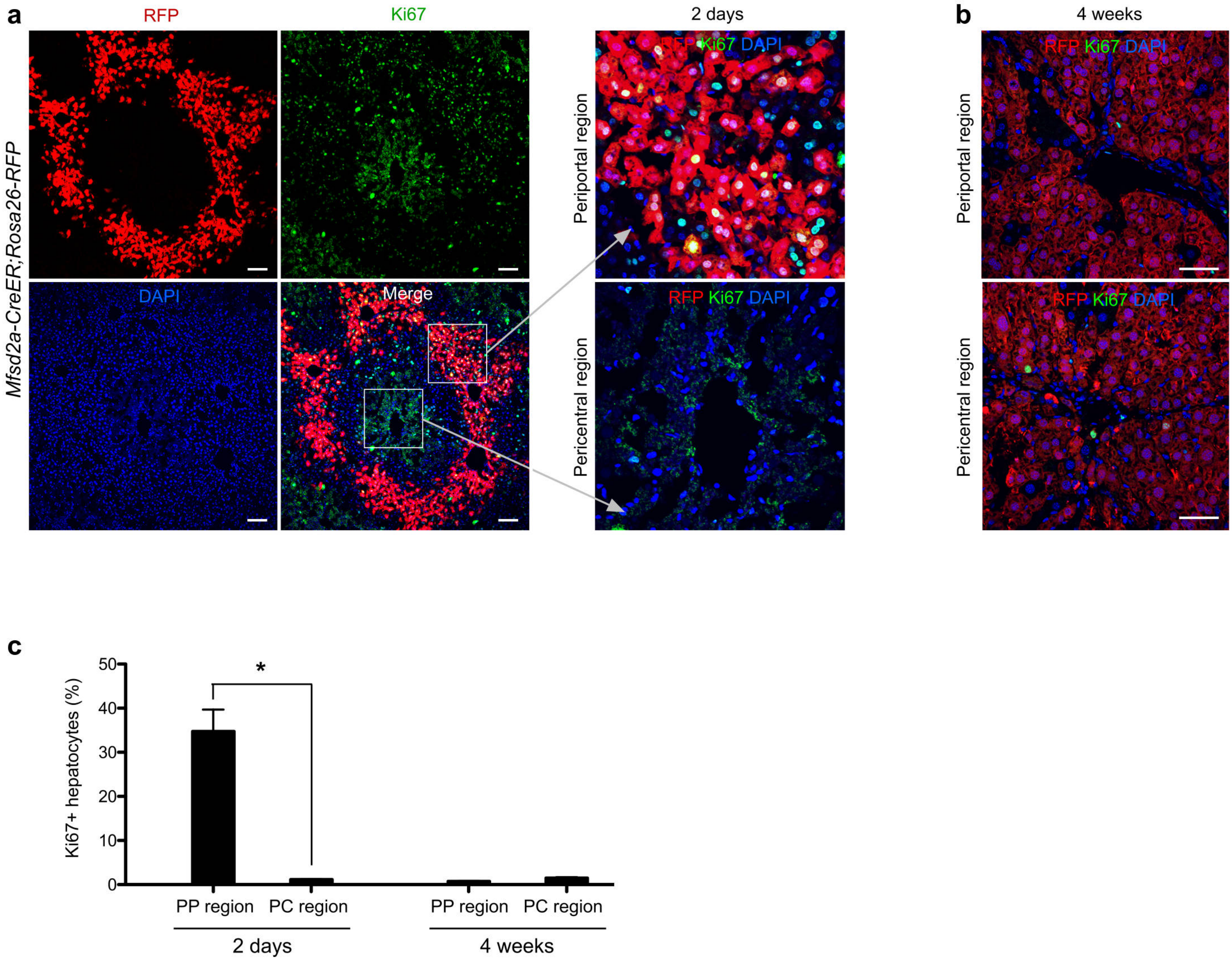

Supplementary Figure 8. Proliferation of hepatocytes in periportal and pericentral regions at early and late stages after CCl_4 treatment. (**a**,**b**) Immunostaining for RFP and Ki67 on sections of *Mfsd2a-CreER;Rosa26-RFP* liver at day 2 after single CCl_4 injection or week 4 after multiple times of CCl_4 treatment (chronic injury). Scale bars, 100 μm. (**c**) Quantification of percentage of Ki67+ hepatocytes in periportal (PP) or pericentral (PC) regions. *$P < 0.05$; n = 4 mice per group; two tailed unpaired *t*-test. Each image is a representative of 4 individual samples.

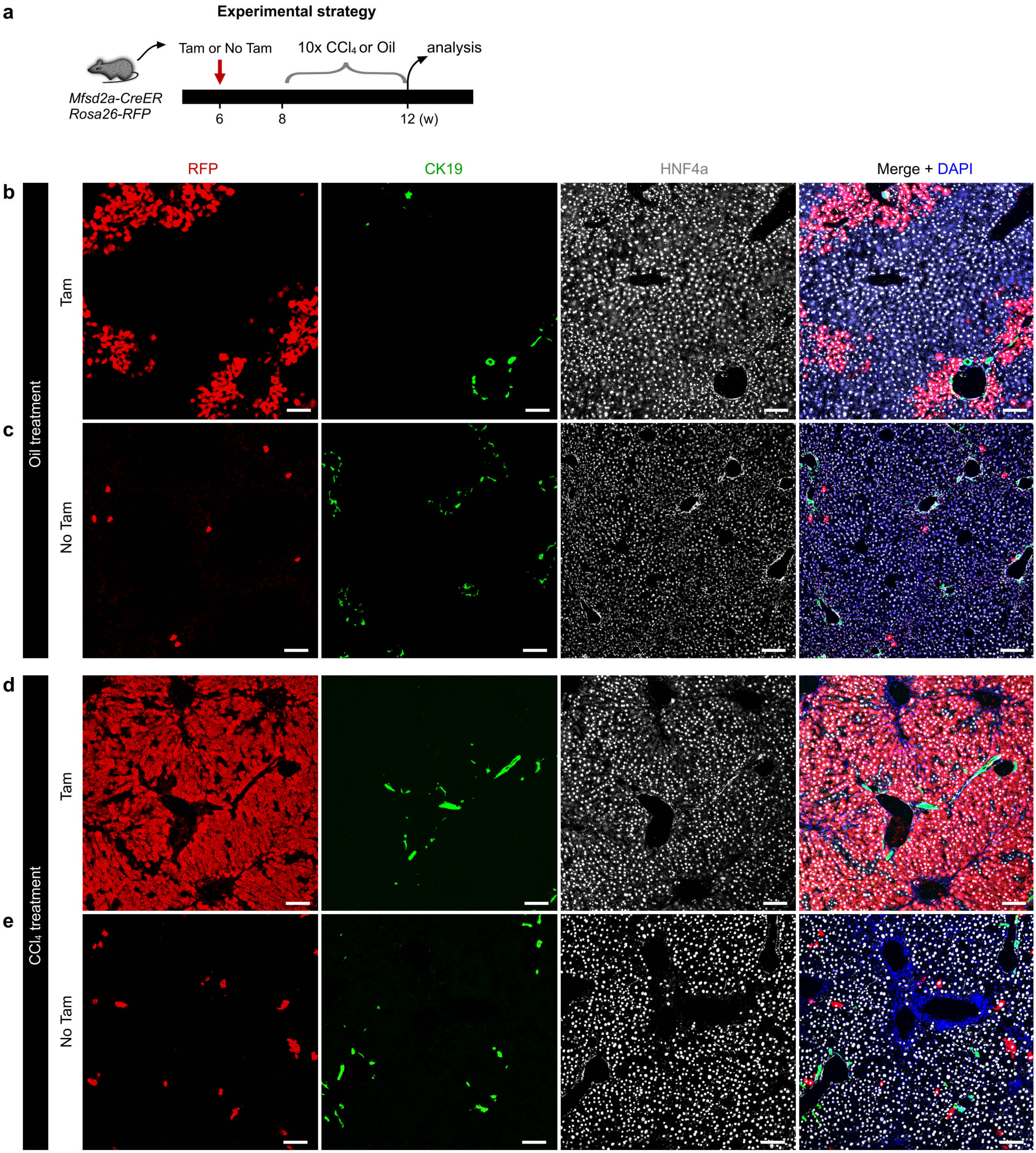

Supplementary Figure 9. Experimental controls for CCl_4 and tamoxifen treatment. (**a**) Schematic figure showing strategies for tamoxifen labeling, CCl_4 treatment and analysis. No Tam, no tamoxifen treatment; Oil, oil treatment as control for CCl_4. (**b-e**) Immunostaining for RFP, CK19 and HNF4a on liver sections from different experiment groups. Scale bars, 100 µm. Each image is a representative of 4 individual samples.

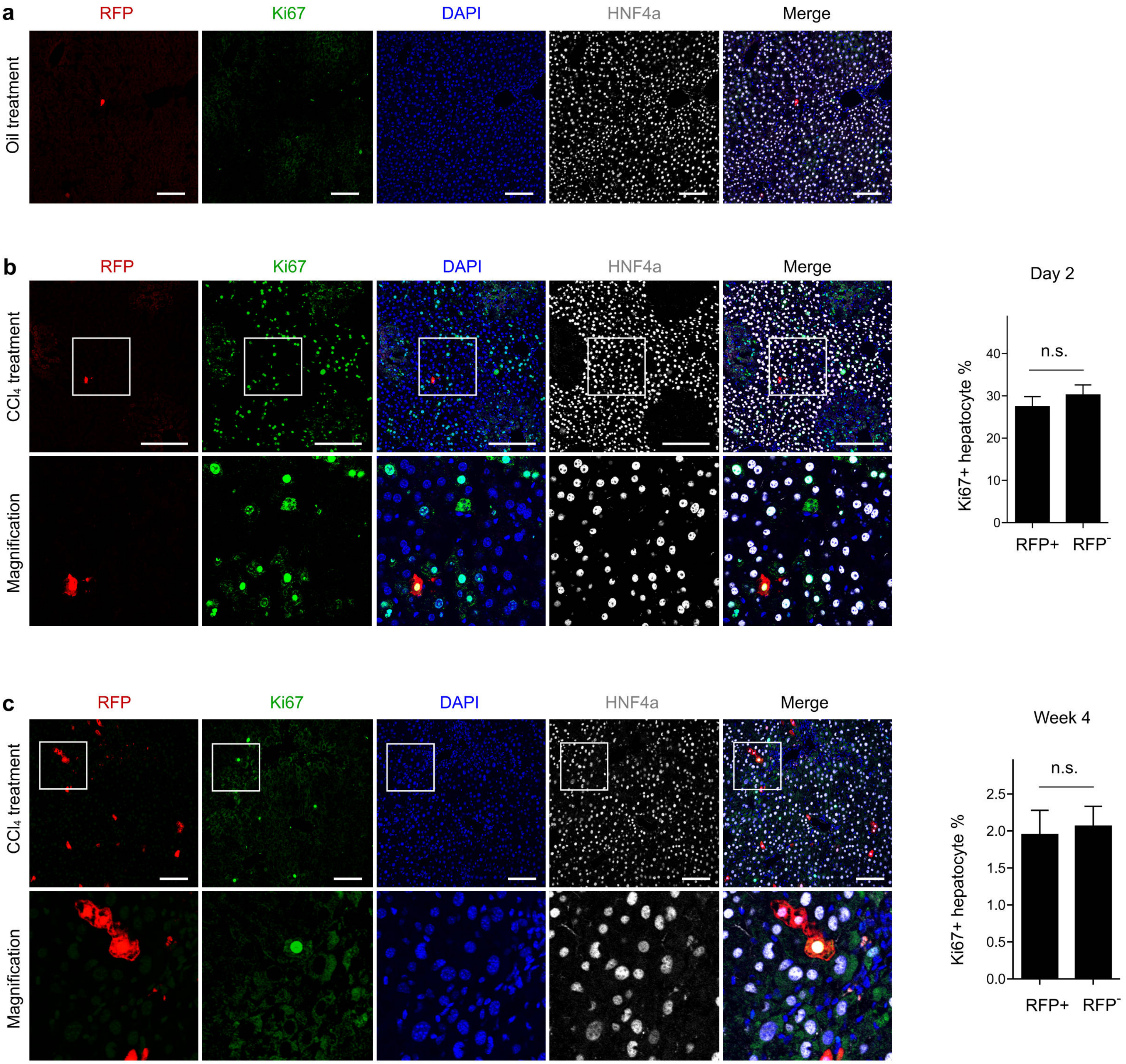

Supplementary Figure 10. Proliferation of RFP+ hepatocytes in *Mfsd2a-CreER;Rosa26-RFP* mice without tamoxifen treatment. (**a**) Immunostaining for RFP, Ki67 and HNF4a on *Mfsd2a-CreER;Rosa26-RFP* liver sections from mice without treatment of tamoxifen. (**b,c**) Immunostaining for RFP, Ki67 and HNF4a on liver sections collected at day 2 (b) after one CCl_4 injection or week 4 (c) after chronic CCl_4 treatment. Right panels show quantification of the percentage of Ki67+ cells in RFP+ or RFP- periportal hepatocytes in livers. n.s., non-significant; n = 4; Scale bars, 100 μm. Each image is a representative of 4 individual samples.

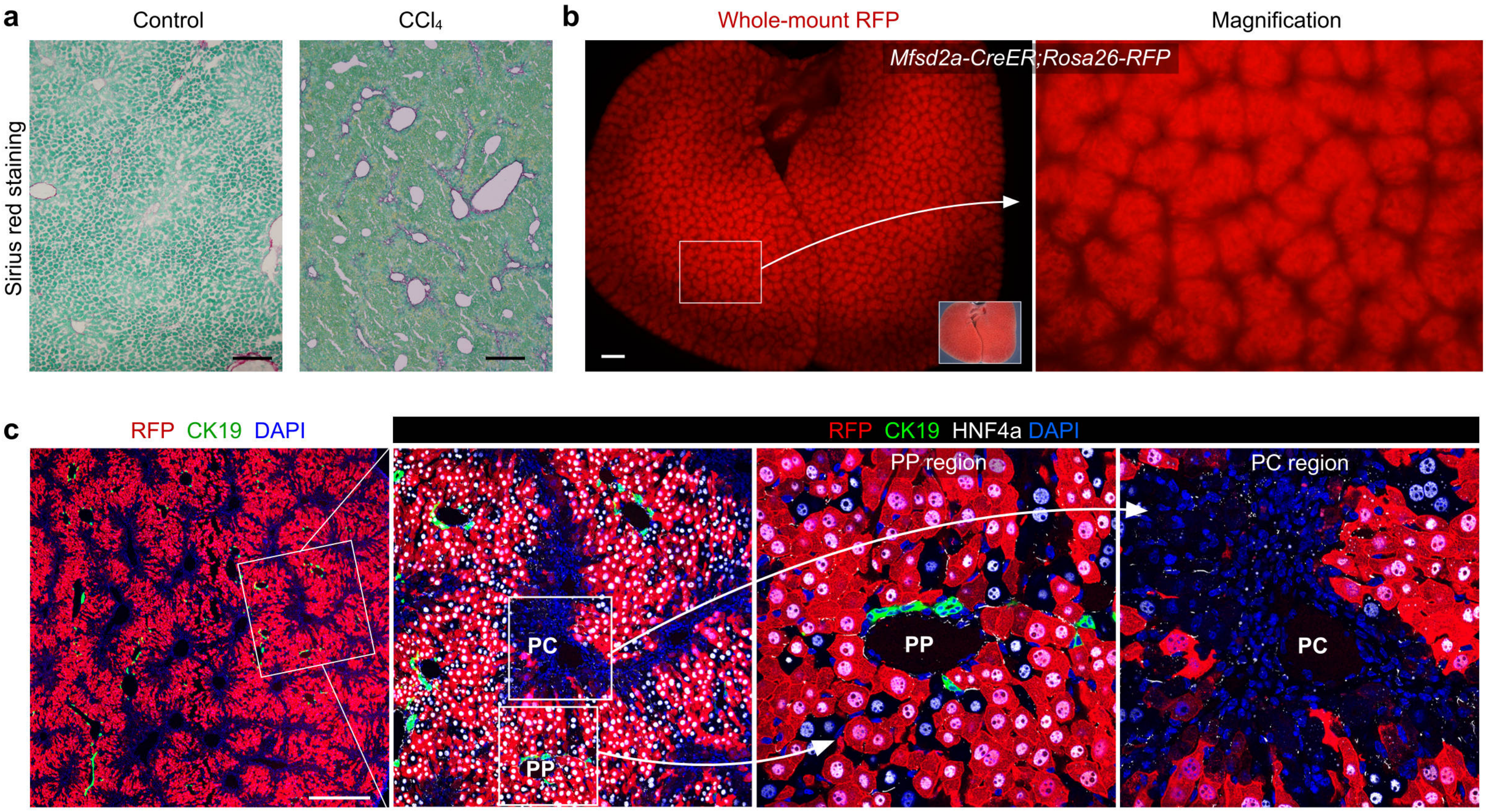

Supplementary Figure 11. Fate mapping of Mfsd2a+ hepatocytes at 2 weeks of CCl_4 treatment. (**a**) Sirius red staining of liver sections from oil (Control) or CCl_4-treated mice (2 weeks). (**b**) Whole-mount fluorescence view of *Mfsd2a-CreER;Rosa26-RFP* liver at 2 weeks of 5 doses of CCl_4 treatment. (**c**) Immunostaining for RFP, CK19 and HNF4a on liver sections showing expansion of PP hepatocytes and reduction of PC hepatocytes during after 2 weeks of CCl_4 induction. Scale bars, 200 μm in **a**; 1 mm in **b**; 500 μm in **c**. Each image is a representative of 4 individual samples.

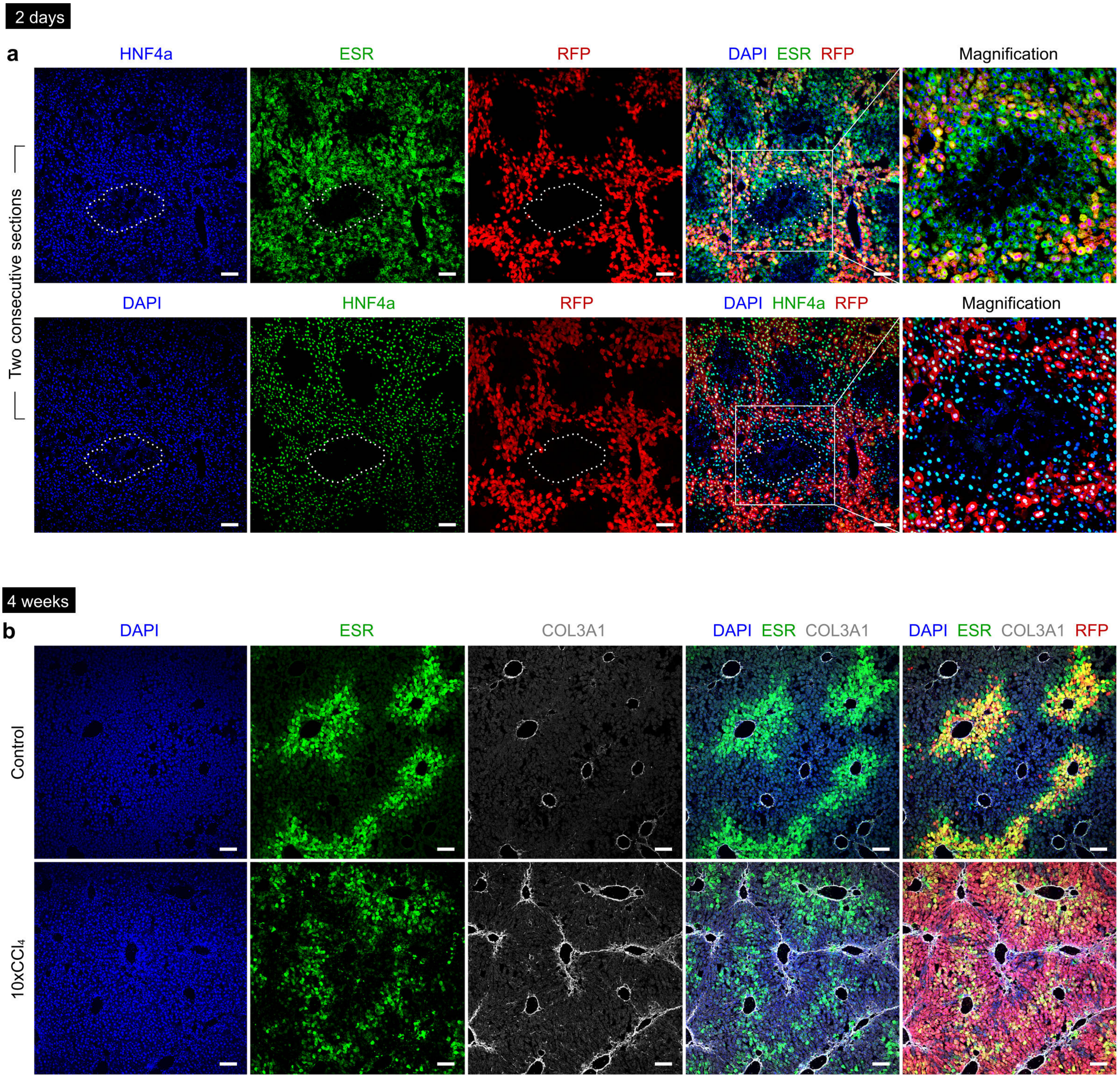

Supplementary Figure 12. Expression of Mfsd2a in early and late stages of injury. (**a**) Immunostaining for ESR as surrogate for Mfsd2a (green, upper panel), HNF4a (green, lower panel), and RFP on consecutive liver sections from *Mfsd2a-CreER;Rosa26-RFP* mice. Tamoxifen was induced at 6 weeks old and mice were treated with CCl_4 at 8 weeks old, and livers were collected 2 days later. (**b**) Immunostaining for ESR, COL3A1 and RFP on Mfsd2a-CreER;Rosa26-RFP liver sections. Tamoxifen was induced at 6 weeks old, and chronic CCl_4 treatment started at 8 weeks old. Samples were collected at 12 weeks old for analysis. Control means oil treatment without addition of CCl_4. Scale bars, 100 μm. Each image is a representative of 4 individual samples.

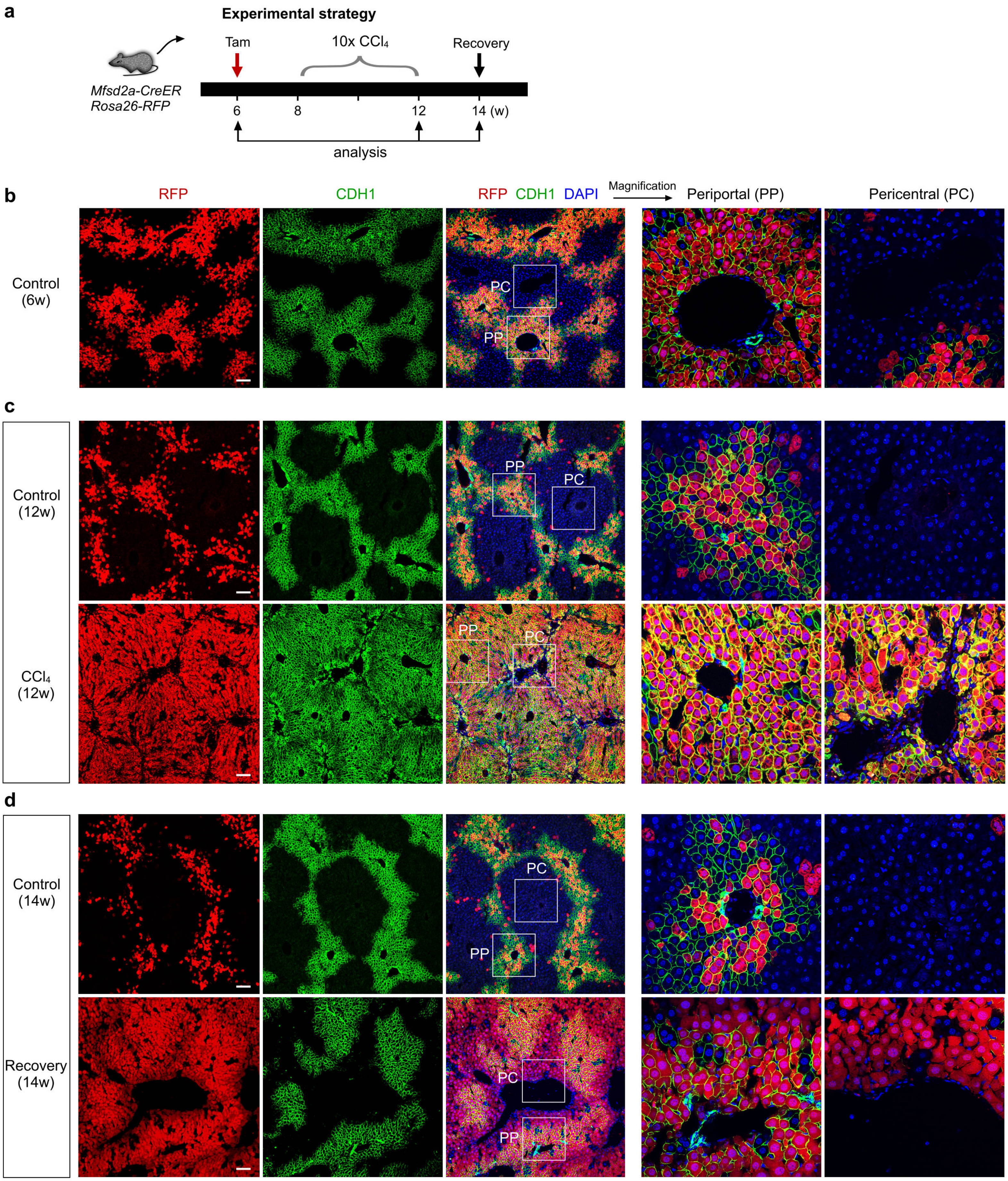

Supplementary Figure 13. Dynamic E-cadherin expression during injury and regeneration. (**a**) A schematic figure showing strategies for tamoxifen administration (Tam), injury induction by CCl_4 and recovery. (**b-d**) Immunostaining for RFP and E-cadherin (CDH1) on liver sections from *Mfsd2a-CreER;Rosa26-RFP* mice at 6 weeks (6w), 12w (after CCl_4 treatment) and 14w (after two weeks recovery) stages. PP, periportal region; PC, pericentral region. Scale bars, 100 µm. Each image is a representative of 4 individual samples.

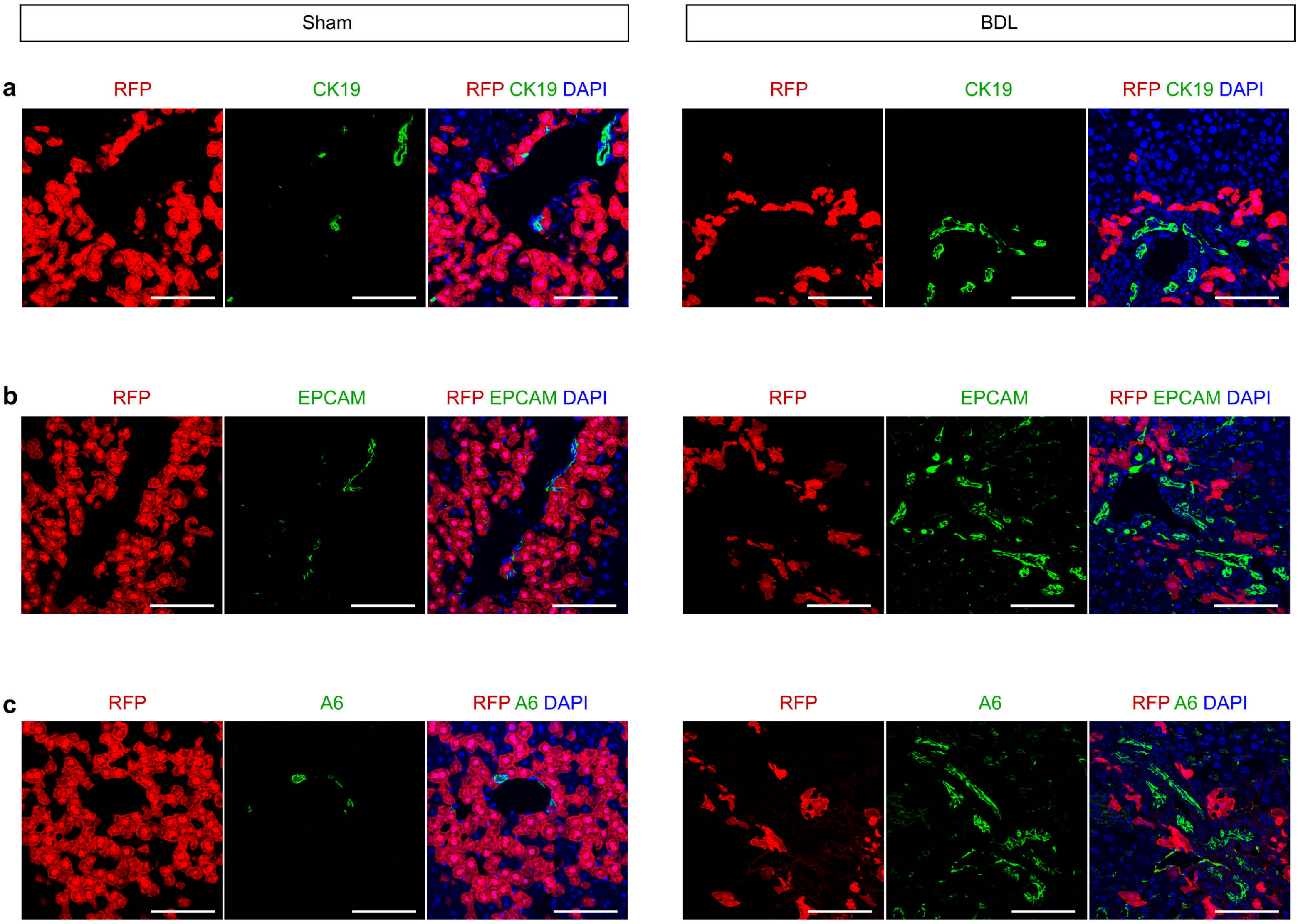

Supplementary Figure 14. Ductal reaction after injury by bile duct ligation (BDL). (a-c) Immunostaining for RFP and CK19 (a), EPCAM (b), A6 (c) on liver sections of *Mfsd2a-CreER;Rosa26-RFP* mice after sham or BDL operations. Scale bars, 100 µm. Each image is a representative of 4 individual samples.

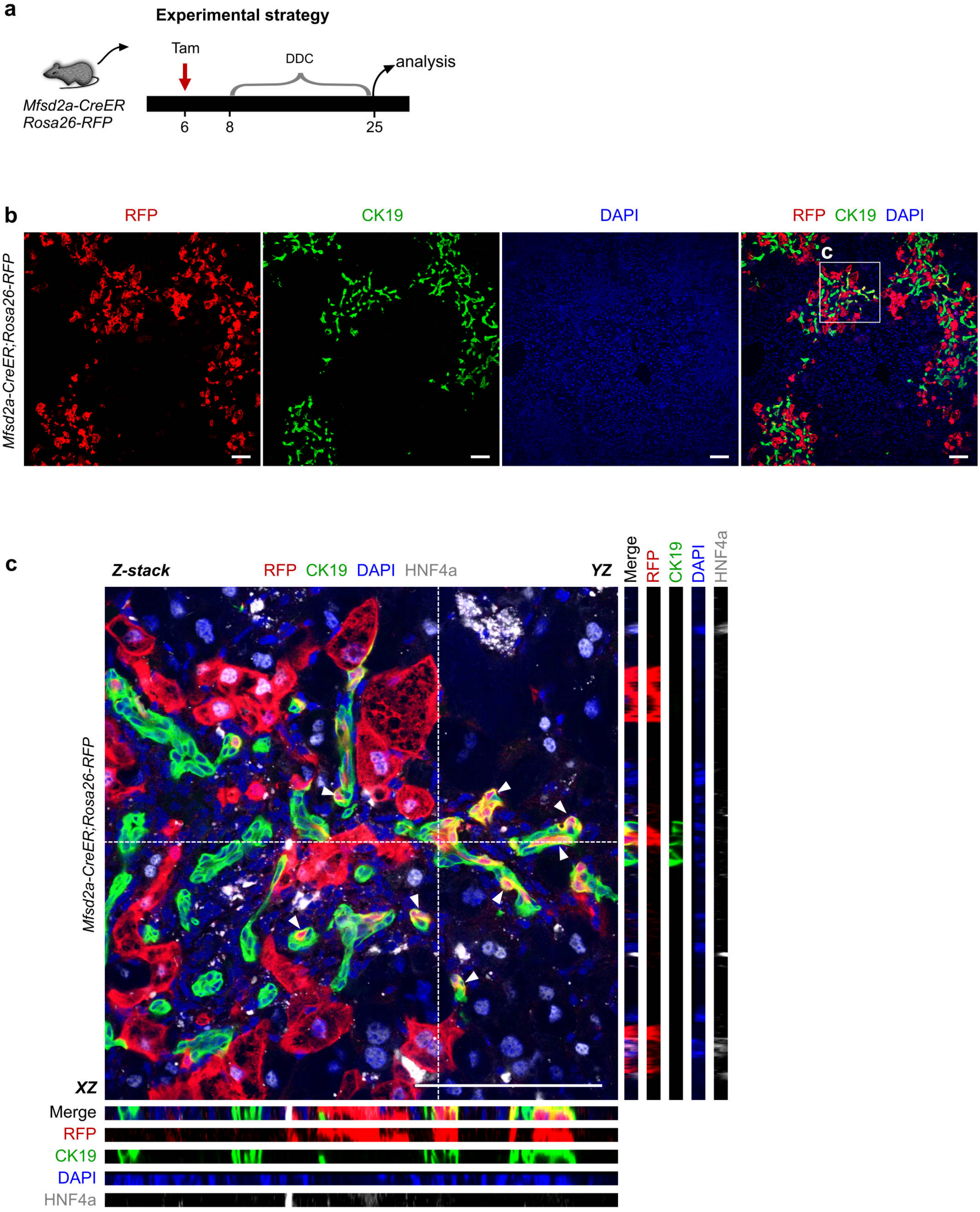

Supplementary Figure 15. Periportal Mfsd2a+ hepatocytes contribute to CK19+HNF4a- biliary epithelial cells. (**a**) Schematic figure showing strategies for tamoxifen induction (Tam) and DDC treatment. (**b**) Immunostaining for RFP and CK19 on liver sections of *Mfsd2a-CreER;Rosa26-RFP* mice treated with DDC. Boxed region is magnified in c. (**c**) Z-stack image of magnified region showed RFP+CK19+HNF4a- biliary epithelial cells (arrowheads). *XZ* and *YZ* indicate signals from dotted lines on *Z*-stack images. Scale bars, 100 μm. Each image is a representative of 4 individual

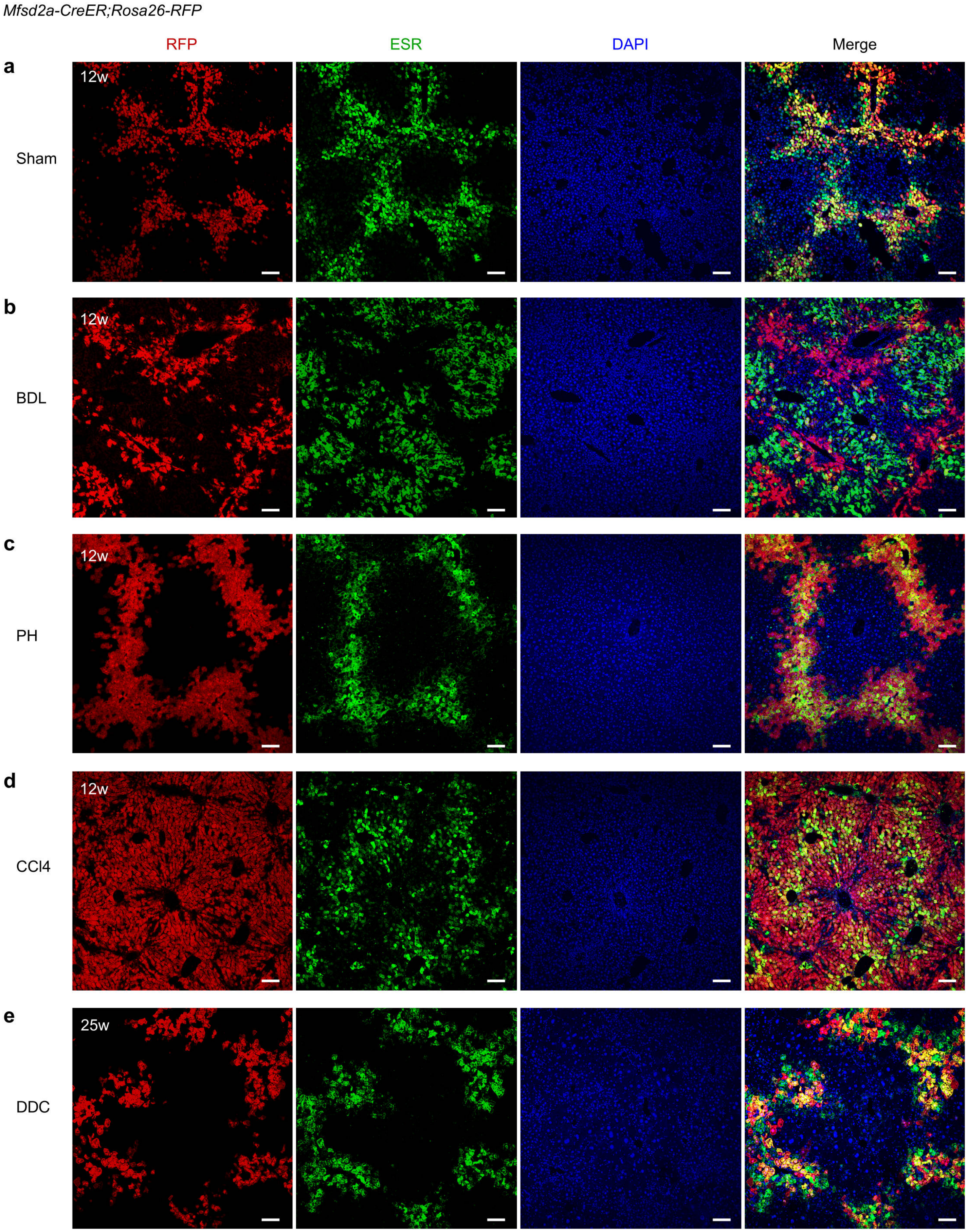

Supplementary Figure 16. Expression of Mfsd2a in sham and injured livers. (**a-e**) Immunostaining for ESR as surrogate for Mfsd2a and RFP on *Mfsd2a-CreER;Rosa26-RFP* mice. Livers in sham, BDL, PH, CCl_4 and DDC groups were collected from mice at the indicated ages. Mice were treated with tamoxifen at 6 weeks old, and objected to injury or sham operation at 8 weeks. Scale bars, 100 μm. Each image is a representative of 4 individual samples.

Supplementary Table 1. Primer sequences used in *in situ* hybiridzation

Gene	Forward	Reverse
Arg1	GACTAGTGAAGAATGGAAGAGTCAGTGTGGTG	ATAAGAATGCGGCCGCTTGGGAGGAGAAGGCGTTTG
Gls2	GACTAGTTGAACAAGATGGCTGGGAACG	ATAAGAATGCGGCCGCTAGGGCTGTGCGGGAATCATAGTC
Pck1	GACTAGTCAGCCAGTGCCCCATTATTG	ATAAGAATGCGGCCGCGGATTCCTGAGTGACCTTGAAGTG
Rnase4	GGAATTCTCCTTGCTTCTGCTCTTGGTGC	ATAAGAATGCGGCCGCACCTTTTGTGAGTAGCCCGTCC
Cyp2e1	GGAATTCGGTTCTTGGCATCACCGTTG	ATAAGAATGCGGCCGCGCTGTGTTTTTCCTTCTCCATCTC
Cyp1a2	GGAATTC CGAGAACTACAAAGACAATGGCGG	ATAAGAATGCGGCCGC CCTAATGAGTATGGGTTATGGGGTG
GS	GACTAGTAACATCAACGACTTTTCTGCCG	ATAAGAATGCGGCCGCCCAGCCAACTCATACTTCTAACTCG
Oat	GACTAGTTGCCACAAAGAAGACAGAGCAAG	ATAAGAATGCGGCCGCTGGTCAGCATTATCTCATCGTCAC

Supplementary Table 2. Primer sequences used in qRT-PCR

Gene	Forward	Reverse
Gapdh	TTGTCTCCTGCGACTTCAAC	GTCATACCAGGAAATGAGCTTG
RFP	ACCTGGTGGAGTTCAAGACC	GTGATGTCCAGCTTGGTGTC
Mfsd2a	ACCTGAAACACCCTCACTCC	GTTGGCAAAGTCGAGACTGA
Arg1	AGACCACAGTCTGGCAGTTG	CCACCCAAATGACACATAGG
Gls2	AATGCCACATTCCAGTCAGA	TAACCTCCACAGAGCACAGC
Pck1	TTTGTAGGAGCAGCCATGAG	TGATGATCTTGCCCTTGTGT
CDH1	CTGGGCAGAGTGAGATTTGA	CCACTTTGAATCGGGAGTCT
Aldh1b1	AACCGCCTAGCTGATCTTGT	TGGAAAGGTTTCCCGTTATC
Sds	GAAGTGGTGGGAGAGATGCT	ATGAGAGGGTCATCAAAGGG
Oat	GGCTGTGGATCATGAGAATG	GCACTGCAGACACAGGGTAT
Cyp2a4	CCAGATATTGAGGCCAAGGT	CAGGTCTGCAAATCTCTGGA
Cyp1a2	ACATCTTTGGAGCTGGCTTT	GCTCCTCATGGATCTTCCTC
Rnase4	CAGGATCGAATGTACCAACG	CGTTTGCACTGGACAGAAGT
Cyp2e1	TAACCAAGTTGGCAAAGCG	CGGCCAGAGAACTCATTCTT
GS	CAGGCTGCCATACCAACTT	TGCACTTCAGACCATTCTCC
Rdh9	CCTTGAGCTACCTGCCTACC	TTCTCAGGCTTCAGGGAAGT
Cyp7a1	GCATCTCAAGCAAACACCAT	TTCTTCAGAGGCTGCTTTCA
Rhbg	CTTCAGCAGTGTGGGCTTTA	AGCGTTGATCAAACTCTCCA
Gstm3	AGCCCTCCCTAGAGATACCC	GGCAGCTCCTTAAACAGGAA
Serpine2	CAAAGAAACGGACATTCGTG	TTGTACCATAAGCCATTCGG
Avpr1	CGTCCAGATGTGGTCAGTCT	GATCGTGGTGGAAGGGTTT
